# Physical Activity, Nutritional Status, and Health-Related Quality of Life in Newly Diagnosed Cancer Patients: Evidence from the NUTRISCREEN Project

**DOI:** 10.3390/nu18050844

**Published:** 2026-03-05

**Authors:** Giuseppe Porciello, Anna Crispo, Francesco Pio Maria Di Carlo, Paola Rocco, Assunta Luongo, Natalia Russo, Elvira Palumbo, Sara Vitale, Sergio Coluccia, Melania Prete, Teresa Di Lauro, Ludovica Abbadessa, Annabella Di Martino, Anna Licia Mozzillo, Emanuela Racca, Arianna Piccirillo, Vittoria Di Giacomo, Maria D’Amico, Martina Fontana, Livia S. A. Augustin, Davide D’Errico, Elisabetta Coppola, Tiziana Stallone, Piera Maiolino, Ileana Parascandolo, Valeria Turrà, Sandro Pignata

**Affiliations:** 1 Epidemiology and Biostatistics Unit, Istituto Nazionale Tumori IRCCS “Fondazione G. Pascale”, 80131 Naples, Italy; g.porciello@istitutotumori.na.it (G.P.); francescopio.dicarlo@istitutotumori.na.it (F.P.M.D.C.); paola.rocco@istitutotumori.na.it (P.R.); natalia.russo@istitutotumori.na.it (N.R.); elvira.palumbo@istitutotumori.na.it (E.P.); sara.vitale@istitutotumori.na.it (S.V.); sergio.coluccia@istitutotumori.na.it (S.C.); melania.prete@istitutotumori.na.it (M.P.); l.augustin@istitutotumori.na.it (L.S.A.A.); 2 Department of Urology and Gynecology, Istituto Nazionale Tumori IRCCS “Fondazione G. Pascale”, 80131 Naples, Italy; teresa.dilauro@istitutotumori.na.it (T.D.L.); davide.derrico@istitutotumori.na.it (D.D.); elisabetta.coppola@istitutotumori.na.it (E.C.); s.pignata@istitutotumori.na.it (S.P.); 3 Dietetics and Artificial Nutrition, Istituto Nazionale Tumori IRCCS “Fondazione G. Pascale”, 80131 Naples, Italy; ludovica.abbadessa@istitutotumori.na.it (L.A.); annabella.dimartino@istitutotumori.na.it (A.D.M.); vittoria.digiacomo@istitutotumori.na.it (V.D.G.); martina.fontana@istitutotumori.na.it (M.F.); ileana.parascandolo@istitutotumori.na.it (I.P.); v.turra@istitutotumori.na.it (V.T.); 4 Melanoma Cancer Immunotherapy and Innovative Therapy Unit, Istituto Nazionale Tumori IRCCS “Fondazione G. Pascale”, 80131 Naples, Italy; annalicia.mozzillo@istitutotumori.na.it; 5 Clinical Sperimental Abdominal Oncology Unit, Istituto Nazionale Tumori IRCCS “Fondazione G. Pascale”, 80131 Naples, Italy; emanuela.racca@istitutotumori.na.it; 6 Otolaryngology and Maxillo-Facial Surgery Unit, Istituto Nazionale Tumori IRCCS “Fondazione G. Pascale”, 80131 Naples, Italy; arianna.piccirillo@istitutotumori.na.it; 7 Colorectal Surgical Oncology, Abdominal Oncology Department, Istituto Nazionale Tumori IRCCS “Fondazione G. Pascale”, 80131 Naples, Italy; m.damico@istitutotumori.na.it; 8 Ente Nazionale di Previdenza e Assistenza a Favore dei Biologi (ENPAB), 00153 Rome, Italy; tiziana.stallone@gmail.com; 9 Pharmacy Unit, Istituto Nazionale Tumori IRCCS “Fondazione G. Pascale”, 80131 Naples, Italy; p.maiolino@istitutotumori.na.it

**Keywords:** physical activity, cancer, health-related quality of life, nutritional assessment, body composition

## Abstract

**Background/Objectives**: Cancer and their treatments could impact physical, nutritional, and psychological health, negatively influencing overall well-being. Accordingly, Health-Related Quality of Life (HRQoL) could be influenced by lifestyle habits, such as physical activity. This study aimed to assess physical activity levels in patients with a primary cancer diagnosis and their association with HRQoL at the first nutritional assessment. **Methods:** Data from the NUTRISCREEN project, part of the ONCOCAMP study (ClinicalTrials.gov ID: NCT06270602), were analyzed. Nutritional and sarcopenia risk, anthropometry and body composition parameters were collected. HRQoL and physical activity (as MET levels) were assessed through validated questionnaires. Descriptive statistics summarized categorical and continuous variables, and multivariable ordinal logistic regression models were performed. **Results:** Nutritional and sarcopenia risk decreased progressively with higher MET levels (*p* = 0.005 and *p* < 0.001, respectively). Adjusted multivariable models showed that HRQoL functional scores improved with increasing MET levels, with significant positive trends for physical (*p* < 0.001), role (*p* < 0.001), emotional (*p* = 0.003), and social functioning (*p* = 0.001), and global health status (*p* < 0.001). Conversely, symptom burden, including fatigue, nausea and vomiting, pain, dyspnea, insomnia, appetite loss, and constipation, decreased across MET quartiles (all *p* < 0.05). **Conclusions:** Overall, our findings suggest that physical activity may positively influence HRQoL among cancer patients. Early assessment helps to identify patients at risk of inactivity and support tailored rehabilitation programs to promote active lifestyles, preserve muscle mass, improve outcomes and overall health status.

## 1. Introduction

Cancer is a significant global health and socioeconomic issue, with a rising incidence. In 2022, there were approximately 20 million new cancer cases and 9.7 million cancer-related deaths worldwide. Looking forward, demographic projections estimate that the number of new cancer cases could rise to 35 million by 2050 [1]. Currently, ongoing advances in early detection and treatment have improved and are expected to further increase survival rates, leading to a growing prevalence of cancer survivors [1]. Lifestyle factors are widely recognized as important determinants of cancer survival. Improved prognosis and long-term survivorship may be influenced by a healthy diet, regular physical activity (PA), smoking status, alcohol consumption, excessive sun exposure, as well as adequate vitamin D supplementation [2]. Among these, PA, defined as any movement that uses skeletal muscles [3], has emerged as a particularly promising area of research in cancer prevention and management during cancer path [4,5,6,7,8]. Numerous studies have demonstrated that higher levels of PA are linked to lower cancer risk, better treatment tolerance, and improved survival across several tumor types [9,10,11,12]. Multiple biological pathways have been proposed to explain this relationship. Exercise can influence sex hormones, including estrogen and testosterone, as well as metabolic hormones such as insulin and insulin-like growth factors, all of which are implicated in tumorigenesis [13,14]. In addition, regular PA regulates chronic inflammation, a recognized driver of tumor development [15]. Beyond cancer prevention, PA could improve prognosis and Health-Related Quality of Life (HRQoL) in cancer patients [9,10,11]. Structured exercise interventions conducted before oncological surgery have been shown to enhance functional capacity, reduce postoperative complications, and accelerate recovery, with potential benefits for long-term outcomes [16,17]. During chemotherapy and endocrine therapy, PA can reduce several common adverse effects, including cancer-related fatigue, peripheral neuropathy (PN), and cardiotoxicity [18]. Similar benefits have been described in patients undergoing radiation therapy, where PA reduces fatigue and improves HRQoL, and early preclinical findings indicate that exercise may also increase tumor radiosensitivity, potentially strengthening treatment responses [19]. Emerging evidence highlights the immunomodulatory effects of exercise. It can enhance immune-cell mobilization, cytotoxicity, differentiation, and trafficking, suggesting a potential way to improve responses to immune checkpoint inhibitors (ICIs). This interaction could strengthen immunotherapy outcomes in cancer patients [20]. In this context, PA has consistently proven safe and feasible throughout cancer treatment. When initiated prior to therapy, PA enhances physical fitness, improves clinical outcomes, and promotes faster recovery [21,22,23].

Beyond these biological mechanisms, it is important to recognize that a cancer diagnosis exerts a profound impact on patients’ overall well-being. A diagnosis is commonly associated with significant psychological distress, including fear, uncertainty, sadness, and anxiety [24]. Disease progression and treatment-related adverse effects can further compromise physical functioning and limit daily activities [25]. These factors can substantially impair patients’ quality of life, a multidimensional construct defined by the World Health Organization (WHO) as an individual’s perception of their position in life within their cultural and value systems, and in relation to personal goals, expectations, standards, and concerns. [26]. Indeed, HRQoL has been consistently identified as an important prognostic indicator in cancer patients [27]. In this context physical exercise appears to have beneficial effects on lifespan, helping cancer patients to manage the side effects of treatment by reducing fatigue and depressive symptoms and increasing survival by reducing mortality rates and recurrence risk [28,29].

Quality of life is significantly influenced by body composition (BC), which is markedly altered by cancer and its therapeutic interventions. Rapid weight loss and changes in Body Mass Index (BMI), reduced appetite, and marked variations in dietary intake, collectively referred to as cancer-associated malnutrition, may significantly impair patients’ quality of life [30,31]. BC alterations also include skeletal muscle mass (SMM) depletion, reductions in muscle strength (MS), and metabolic dysregulation, that compromise physical performance, exacerbate fatigue, and restrict the capacity to perform activities of daily living, thereby further impairing overall functional status and well-being [32]. When concomitant, these alterations characterize sarcopenia, which has emerged as a relevant pan-cancer prognostic biomarker [33,34]. Engagement in regular PA positively influences BC, increasing muscle mass and quality, and thereby supporting higher levels of physical fitness [35]. Structured exercise interventions can beneficially modulate BC and related physiological parameters, including muscle and fat compartments, metabolic function, and circulating cytokines, with effects observed independently of prior chemotherapy exposure [36]. Exercise interventions reduce treatment-related fatigue, enhance physical fitness, and positively affect BC and biomarkers, with low-to-moderate intensity recommended during treatment and moderate-to-high intensity beneficial after therapy [37]. Taken together, these observations underscore the complex interplay between PA, nutritional status, and quality of life, highlighting the need for integrated assessment and intervention in cancer care.

The period immediately following cancer diagnosis represents a clinically and psychologically critical phase, marked by emotional distress, behavioral changes, and potential early decline in nutritional and functional status [38]. While particularly vulnerable, this window also offers an opportunity for early assessment of modifiable risk factors.

In this context, a clearer understanding of PA levels and HRQoL at the time of first nutritional evaluation becomes essential. Through its positive effects on muscle mass, strength, and overall BC, PA contributes to improved daily functioning and well-being, highlighting its integral role in maintaining quality of life in cancer patients. Our cross-sectional analysis aimed to investigate PA levels in newly diagnosed cancer patients and their correlation with multiple dimensions of HRQoL. The objective was to determine whether higher levels of PA at the time of diagnosis are associated with more favorable quality of life profiles, thereby supporting the development of targeted early interventions. In addition, the study explored gender-specific differences in nutritional assessment parameters and PA patterns, with the goal of identifying distinctive needs that may guide more personalized approaches to care.

## 2. Materials and Methods

### 2.1. Background

Data shown are part of the NUTritional and Sarcopenia RIsk SCREENing (NUTRISCREEN) project, which involved the INT IRCCS “Fondazione G. Pascale” of Naples and previously Ente Nazionale di Previdenza e Assistenza a Favore dei Biologi—ENPAB. This project is part of the ONCOCAMP research protocol, a retrospective–prospective multicenter observational study (ClinicalTrials.gov ID: NCT06270602, registered on 21 February 2024), aimed at improving the appropriateness, effectiveness, and safety of diagnostic and therapeutic pathways, as well as clinical strategies and procedures within the Campania Oncology Network (“The ONCOCAMP Study”). In this context, the NUTRISCREEN project aims to identify early malnutrition and sarcopenia risks in cancer patients using validated assessment tools, enabling targeted interventions and strategies to improve patient quality of life and prognosis [30].

### 2.2. Data Collection

Data from patients enrolled between July 2023 and July 2025 were collected during their first nutritional visit at our cancer center using a customized Case Report Form (CRF). Specifically, this analysis included patients with a first diagnosis of cancer who underwent nutritional screening; patients with metastatic disease, recurrence and those with missing data were excluded. Variables considered were gender, age, marital status (single, married or cohabiting, divorced or separated, widowed), education level (primary, lower secondary, high school, university or postgraduate), smoking status (current smoker, former smoker, never smoker), and presence of intermediate risk factors or comorbidities. Patients’ cancers were classified into the following categories: breast, gastrointestinal, head and neck, lung, skin, and uro-gynecological. Gastrointestinal cancers include esophagus, stomach, pancreas, liver, gallbladder, and rectum. Uro-gynecological cancers included bladder, prostate, kidney, testis, vulva, cervix, endometrium, ovary, and uterus.

### 2.3. Nutritional and Sarcopenia Risk Screening Tools

To assess malnutrition and sarcopenia risk in our population, the Nutritional Risk Screening 2002 (NRS-2002) and the Strength, Assistance with walking, Rise from a chair, Climb stairs, and Falls (SARC-F) [34,39], were administered.

The NRS-2002 includes two main components: a short set of four questions to quickly identify patients who may be at nutritional risk (pre-screening). If the answer to any of these questions is “yes”, the full screening is conducted. Final screening involves scoring the severity of impaired nutritional status, based on percentage of recent weight loss, reduction in food intake, BMI, disease severity and age which reflects increased nutritional requirements due to the underlying illness. The total score ranges from 0 to 7. A score of ≥3 indicates nutritional risk, and the patient should receive a full nutritional assessment and appropriate support. NRS-2002 is widely recommended because of its simplicity, clinical applicability, and evidence-based structure. By assessing both nutritional status and disease severity, it is particularly useful in oncology settings, where patients often face systemic inflammation and heightened metabolic demands.

The SARC-F is a rapid, practical, and non-invasive tool that consists of five questions assessing muscle strength, physical performance, and functional ability, each with three possible responses: “No difficulty”, “Some difficulty”, or “Severe difficulty or inability to perform the task”. For each of these responses, a score of 0, 1, or 2, was assigned, respectively. Total score ranges from 0 to 10, with higher score reflecting an increased risk of sarcopenia. A score of ≥4 suggests a risk of sarcopenia and indicates the need for further diagnostic evaluation, such as muscle strength tests and BC analysis.

### 2.4. Measurement of Physical Activity in Cancer Patients

PA was assessed using the International Physical Activity Questionnaire—Short Form (IPAQ-SF) [40,41,42], a validated 7-item self-report instrument designed to capture PA over the previous 7 days in adults aged 15–69 years. The questionnaire records the frequency (days/week) and duration (minutes/day) of activities performed for at least 10 continuous minutes at three intensity levels: vigorous, moderate, and walking as well as time spent sitting on a typical weekday. Vigorous activities are defined as those requiring hard physical effort and causing substantially increased breathing (e.g., fast cycling, aerobics), whereas moderate activities include tasks inducing a moderate increase in breathing (e.g., carrying light loads, recreational cycling). Walking items encompass all walking performed for work, transport, household tasks, or leisure.

PA was assessed according to type, frequency, duration, and intensity. Intensity was expressed using Metabolic Equivalents (METs), which quantify energy expenditure relative to a person’s resting metabolic rate. One Metabolic Equivalent (MET) represents the energy expenditure at rest (3.5 mL O_2_/kg/min) and allows the energy cost of PA to be expressed as multiples of resting metabolic rate. METs provide a practical method to classify activity intensity, compare different exercises, and estimate functional capacity or exercise tolerance. They are widely used to quantify energy expenditure in both household and recreational activities and to prescribe safe activity levels without exceeding target intensity [43].

Activities were classified as vigorous (≥6 METs; e.g., running, fast cycling), moderate (3–5.9 METs; e.g., brisk walking, vacuuming), or light (<3 METs; e.g., standing, slow walking). Sedentary behavior was defined as any waking behavior with an energy expenditure ≤ 1.5 METs while sitting or reclining. Total PA levels were calculated as the combination of frequency, intensity, and duration across different activity types [44]. In accordance with the IPAQ structure, PA data were derived using standard MET values and subsequently calculated as total MET-minutes per week.

### 2.5. Health-Related Quality of Life Assessment

HRQoL was assessed using the European Organization for Research and Treatment of Cancer Quality of Life Questionnaire (EORTC QLQ-C30, version 3.0), a validated tool specifically designed for oncology patients. The questionnaire comprises 30 items across multiple domains: five functional scales (physical, role, emotional, cognitive, and social), three symptom scales (fatigue, nausea and vomiting, and pain), six single items addressing additional symptoms (dyspnea, sleep disturbance, appetite loss, constipation, diarrhea, and financial impact), and a global health status/quality of life scale (GHS), calculated as the mean of two items evaluating overall health and overall quality of life [45].

Responses are recorded on a four-point Likert scale (from 1 = “Not at all” to 4 = “Very much”), except for the GHS/quality of life scale, which uses a seven-point scale (from 1 = “Very poor” to 7 = “Excellent”). Scores are linearly transformed to a 0–100 scale: higher scores on functional scales and GHS indicate better functioning and quality of life, whereas higher scores on symptom scales indicate greater symptom burden.

The EORTC QLQ-C30 Summary Score (SumSc) was calculated as the mean of 13 QLQ-C30 scales, excluding the GHS and financial impact item. Symptom scales were reverse-coded prior to calculation to ensure a uniform score direction. SumSc was computed only when all 13 scales were available, in accordance with the EORTC QLQ-C30 Scoring Manual, using scale scores based on at least 50% of completed items per scale. To obtain a single composite measure of overall functioning and symptom burden, mean functional and symptom scores were calculated and denominated as the Functional Score and Symptoms Score, respectively [46].

### 2.6. Body Composition and Dietary Assessment

Patients’ nutritional assessment included anthropometric measurements, BC analysis, and diet quality evaluation. The main anthropometric parameters collected were body weight, BMI, Waist Circumference (WC), and Hip circumference (HC). Body weight and height were measured using a calibrated mobile scale equipped with a stadiometer (WUNDER C201, Wunder Sa.Bi. srl, Milan, Italy) while circumferences were assessed using a non-elastic measuring tape (SECA 201; SECA, Hamburg, Germany). All measurements were performed in triplicate to minimize measurement error and following standardized procedures.

BC was assessed using bioelectrical impedance analysis (BIA) with the Akern BIA 101 BIVA^®^ PRO device (Akern Srl, Pontassieve, Italy) at a frequency of 50 Hz, with electrodes placed on the hands and feet. Measurements were performed under standardized conditions, in the morning, with participants fasting and in a supine position to ensure consistency and accuracy. Information regarding recent fluid intake was collected, and potential confounders such as edema, prostheses, or other clinical conditions affecting impedance measurements were carefully evaluated. This method provided estimates of key BC components, including fat mass (FM), fat-free mass (FFM), body cell mass (BCM), SMM, appendicular skeletal muscle mass (ASMM), and the Extracellular water (ECW), Intracellular water ratio (ICW). Phase angle (PhA), a marker of cellular membrane integrity and nutritional status, was calculated as:

PhA (°) = arctan(Xc/R) × (180/π)

where Xc is reactance and R is resistance. Relative indices for muscle and fat mass—Fat mass index (FMI), Fat-free mass index (FFMI), Body cell mass index (BCMI), Skeletal Muscle Index (SMI), and Appendicular Skeletal Muscle Index (ASMI) were provided directly by the device software (Bodygram™ Dashboard V.3.4.9) and adjusted for height squared (m^2^). Anthropometric parameters, including BMI and WC, were also recorded; BMI was calculated as weight (kg) divided by height squared (m^2^), following WHO guidelines. BIA-derived parameters were expressed in kilograms, except ECW/ICW as a ratio, PhA in degrees (°), and R and Xc in ohms (Ω).

Diet quality and adherence to a Mediterranean Diet (MedDiet) were assessed using the validated 17-item PREDIMED questionnaire [47,48]. Although originally developed for general adult populations and not specific to oncology patients, this instrument reliably captures consumption frequency of typical and non-typical Mediterranean foods, including vegetables, fruits, whole grains, alcohol, red and processed meats, and added sugars. The questionnaire was administered electronically to ensure standardized data collection and to minimize missing or incomplete responses. In accordance with guidelines advising against alcohol consumption in cancer patients [49], the item on wine intake was omitted from the administered questionnaire.

### 2.7. Statistical Analysis

Categorical variables were summarized as counts (n) and percentages (%), whereas continuous variables were expressed as mean ± standard deviation (SD): in particular we have used the ± notation for reporting mean values in the main text, while in the tables we report data as mean (SD). In the description of general characteristics, age was reported as mean ± SD, while gender, marital status, educational level, smoking status, cancer type, and comorbidities were presented as counts and percentages. NRS-2002 score, SARC-F score, BMI categories (normal weight, overweight, and obese), and MedDiet adherence score (<7 vs. ≥7) were reported as counts (n) and percentages (%). Continuous variables, such as anthropometric data (WC, HC) BC parameters (including PhA, ECW, ICW, BCMI, FMI, FFMI, SMI, ASMI) and quality of life scores (EORTC QLQ-C30 SumSc, Functional Score, Symptoms Score, GHS, functional scales, and symptom scales) were expressed as mean ± SD. Regarding PA, MET-min/week values were calculated in accordance with the official Guidelines for Data Processing and Analysis of the IPAQ [50], applying the recommended scoring protocol, data-cleaning procedures, and algorithms to account for potential overreporting and related sources of bias. Participants were categorized into quartiles based on total weekly MET-minutes: Low (≤396), Moderate (397–1986), Moderate-to-High (1987–4767), and Intensive (≥4768). Finally, although quality of life items were reported on a numerical scale ranging from 0 to 100, their empirical distributions were discrete, with values clustering at selected points of the scale.

Associations were assessed both overall and stratified by gender using the χ^2^ test for categorical variables and the Kruskal–Wallis test for continuous variables. Adjustment for multiple comparisons was performed using the Benjamini–Hochberg false discovery rate (FDR) method. Based on these distributions, multivariable ordinal logistic regression models were used to evaluate associations between quality of life outcomes and total weekly MET-minute quartiles, NRS-2002, and SARC-F scores. All models were adjusted for age, gender, cancer type, FFMI, and BCMI. The proportional odds assumption was verified for each model. Statistical significance was set at *p* < 0.05. Statistical analyses were performed using R statistical software (version 4.5.2; R Core Team, R Foundation for Statistical Computing, Vienna, Austria, 2025). 

## 3. Results

### 3.1. Descriptive Analysis of Anthropometric, Lifestyle, and Outcome Variables

General characteristics (N = 680) are summarized in Table 1. Most participants were male (57.4%), and 77.7% were married or cohabiting, while only 6.0% were widowed. The mean age was 61.8 ± 12.3 years. Education levels varied: 10.0% had completed only elementary school, 38.2% had finished upper secondary education, and 21.8% held a university or postgraduate degree. Current smokers accounted for 24.4% of the sample, whereas former smokers represented the largest share at 43.2%. Uro-gynecological cancers were the most frequent (30.3%), followed by gastrointestinal cancers (28.2%) and lung cancer (14.6%). Melanoma accounted for 11.3% of cases, breast cancer for 12.1%, while head and neck tumors were less common (3.5%). Most patients had not received neoadjuvant therapy (66.5%), and comorbidities were reported in 59.9% of the cohort.

Participants were generally in good nutritional status (NRS-2002 < 3) and not at risk of sarcopenia (SARC-F < 4), with 85.3% and 83.7%, respectively. BMI categories were relatively balanced, with 39.0% classified as overweight, 34.1% as normal weight, and 26.9% as obese. Mean Waist and Hip circumferences were 96.2 ± 14.0 cm and 102.0 ± 11.4 cm, respectively. BIA showed a mean PhA of 5.6 ± 1.2°, ECW of 19.2 ± 4.2 L, ICW of 20.8 ± 5.2 L, BCMI of 10.1 ± 2.2, FMI of 8.0 ± 3.8, FFMI of 19.6 ± 2.6, SMI of 9.2 ± 1.7, and ASMI of 7.4 ± 1.2.

High adherence to the MedDiet was observed in 66.0% of patients. Overall, patients reported relatively high levels of functioning across the five functional domains, with mean scores ranging from 69.4 ± 25.2 for emotional functioning to 85.1 ± 18.4 for cognitive functioning, indicating generally preserved quality of life. GHS had a mean score of 58.9 ± 24.3, reflecting a moderately positive perception of overall health. Symptom scales indicated that fatigue was the most frequently reported symptom (31.3 ± 25.7), followed by insomnia (31.2 ± 30.1), while nausea and vomiting were less common (7.6 ± 16.0). Participants were evenly distributed across the PA quartiles (MET-minutes/week or MET levels), with 25.3% in the Low, 24.8% in the Moderate, 24.8% in the Moderate-to-High, and 25.1% in the Intensive group.

### 3.2. Associations Between MET Levels and Participant Characteristics, Body Composition, Quality of Life, and Nutritional Status

Patient characteristics according to total weekly MET categories are presented in Table 2. No significant differences were observed across MET categories for age, gender, marital status, education level, comorbidities, BMI categories or smoking status. However, the distribution of cancer types differed significantly (*p* = 0.001), with gastrointestinal cancers being more frequent in the Moderate MET category and uro-gynecological cancers predominating in the Intensive MET category. Both nutritional risk (NRS-2002 ≥ 3) and sarcopenia risk (SARC-F ≥ 4) differed significantly across MET levels (*p* = 0.005 and *p* < 0.001, respectively), showing a progressive decrease in the prevalence of at-risk patients with increasing MET levels.

BIA and quality of life parameters varied across MET levels. Higher MET categories were associated with higher PhA values (*p* < 0.001), whereas no significant differences were observed for ECW, ICW, FM, FFM, or skeletal muscle indices. Quality of life scores improved progressively with increasing MET levels, with higher physical (*p* < 0.001), role (*p* < 0.001), emotional (*p* = 0.003), cognitive (*p* = 0.052), and social functioning (*p* = 0.001), as well as higher GHS (GHS; *p* < 0.001). In contrast, symptom scores including fatigue, nausea and vomiting, pain, dyspnea, insomnia, and appetite loss decreased as MET levels increased (all *p* < 0.05). No significant differences were observed in MedDiet adherence across MET levels.

### 3.3. Associations Between MET Levels and Participant Characteristics by Gender

Detailed results are provided in Supplementary Table S1. No significant differences across MET intensity categories were observed for age, marital status, educational level, smoking status, cancer type, comorbidities, BMI, or MedDiet adherence in either women or men. Male patients at higher risk of malnutrition (NRS-2002 ≥ 3), were more represented in the Low category (*p* < 0.05); the trend for women was similar but did not reach statistical significance. Whereas women at higher risk of sarcopenia (SARC-F ≥ 4) were more commonly classified among the least physically active, for men, this difference was not significant (*p* = 0.134).

PhA was significantly higher in intensity MET level for both genders (*p* = 0.015 in women; *p* = 0.003 in men). In men, a significant higher value in the intensity group was also observed for ICW (*p* = 0.001).

Mean HRQoL scores, including SumSc, Functional Score, and selected functional scale items (physical and role functioning), increased significantly across PA quartiles, from the Low to the Intensive group, for both genders. Among men, emotional, cognitive, social functioning, and GHS scores showed a significant increase across PA quartiles, from Low to Intensive (*p* < 0.001); this was not the case for women. Among men, all symptom scales and the overall Symptom Score decreased significantly from the Low to the Intensive group (generally *p* <0.05). In women, a significant decrease was observed only for fatigue (*p* = 0.009) and pain (*p* = 0.018), while trends for nausea/vomiting and dyspnea did not reach significance.

### 3.4. Multivariable Logistic Regression of Physical Activity, Nutritional and Sarcopenia Risk on Quality of Life

#### 3.4.1. Functional Scales and GHS

HRQoL functional scale scores increased progressively across MET levels, showing a significant positive trend from Low to Intensive levels for all domains except cognitive functioning (*p*-trend ≤ 0.001 for physical, role, emotional, and social functioning, and GHS, *p* = 0.09 for cognitive functioning). Compared with the Low, all higher MET quartiles were associated with progressively higher odds of better functional scores (except for cognitive), with the greatest increase observed in the Intensive quartile (physical OR = 4.97, 95% CI 3.02–8.25; role OR = 5.52, 95% CI 3.21–9.60; emotional OR = 2.06, 95% CI 1.28–3.34; social OR = 2.04, 95% CI 1.23–3.40; GHS OR = 2.65, 95% CI 1.63–4.32).

Patients at nutritional risk (NRS-2002 ≥ 3) had significantly lower odds of reporting higher functional scores in most domains, including physical (OR = 0.35, 95% CI 0.22–0.55; *p* < 0.001), role (OR = 0.33, 95% CI 0.20–0.54; *p* < 0.001), emotional (OR = 0.52, 95% CI 0.32–0.81; *p* < 0.001), and GHS (OR = 0.34, 95% CI 0.21–0.54).

**Figure 1:**
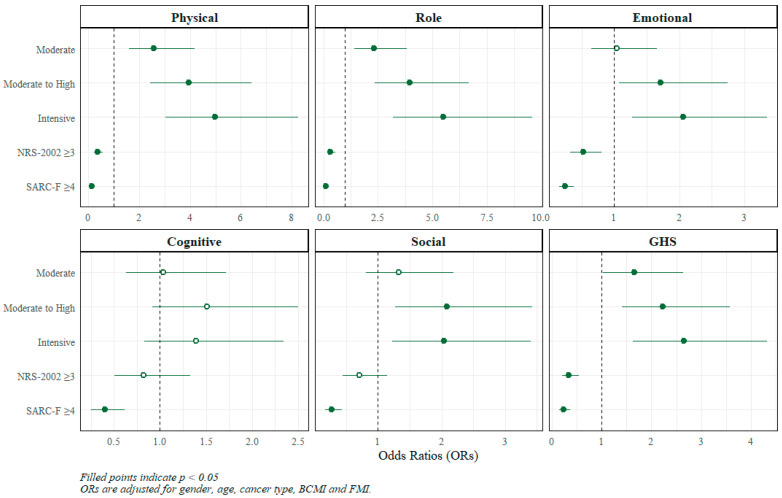
Multivariable-adjusted forest plots for the association of PA levels with functional HRQoL scales. Abbreviations: NRS-2002, Nutritional Risk Screening 2002; SARC-F, Strength, Assistance with walking, Rise from a chair, Climb stairs, and Falls; GHS, Global Health Status.

Higher sarcopenia risk (SARC-F ≥ 4) was associated with substantially lower odds of reporting better functional scores across all domains, including physical (OR = 0.14, 95% CI 0.09–0.23), role (OR = 0.13, 95% CI 0.08–0.21), emotional (OR = 0.24, 95% CI 0.16–0.38), cognitive (OR = 0.40, 95% CI 0.25–0.62), social (OR = 0.27, 95% CI 0.17–0.43), and GHS (OR = 0.24, 95% CI 0.15–0.37). The estimates from the multivariable models were graphically displayed using forest plots (Figure 1). All results are presented in the Supplementary Materials (Table S2).

#### 3.4.2. Symptoms Scales

In contrast to functional scales, symptom burden decreased progressively across MET quartiles, with significant trends observed for most symptoms, including fatigue, nausea and vomiting, pain, dyspnea, insomnia, appetite loss, and constipation (all *p*-trend ≤ 0.043). Patients in the Moderate and Moderate-to-High MET quartiles showed intermediate reductions in the odds of higher symptom scores compared with those in the Low quartile, with the greatest reductions observed in the Intensive group (fatigue OR = 0.23, 95% CI 0.14–0.37; nausea and vomiting OR = 0.40, 95% CI 0.21–0.74; pain OR = 0.31, 95% CI 0.18–0.52; dyspnea OR = 0.37, 95% CI 0.20–0.68; insomnia OR = 0.58, 95% CI 0.35–0.95; appetite loss OR = 0.37, 95% CI 0.20–0.70; diarrhea OR = 0.49, 95% CI 0.24–0.99).

**Figure 2:**
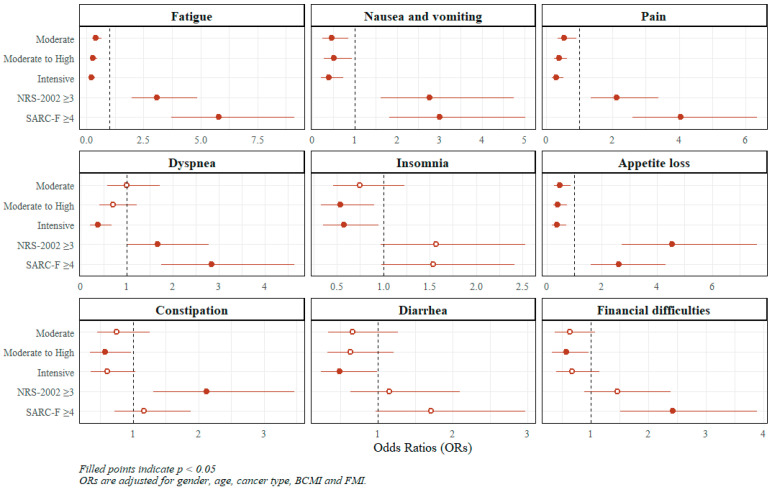
Multivariable-adjusted forest plots for the association of PA levels with symptoms HRQoL scales. Abbreviations: NRS-2002, Nutritional Risk Screening 2002; SARC-F, Strength, Assistance with walking, Rise from a chair, Climb stairs, and Falls.

Patients at nutritional risk (NRS-2002 ≥ 3) had significantly higher odds of experiencing more severe symptoms across most domains, particularly fatigue (OR = 3.09, 95% CI 1.97–4.85), nausea and vomiting (OR = 2.77, 95% CI 1.61–4.75), pain (OR = 2.13, 95% CI 1.35–3.38), dyspnea (OR = 1.68, 95% CI 1.01–2.78), appetite loss (OR = 4.54, 95% CI 2.72–7.61), and constipation (OR = 2.12, 95% CI 1.31–3.46). Similarly, patients at higher sarcopenia risk (SARC-F ≥ 4) experienced even greater increases in symptom severity, most notably for fatigue (OR = 5.80, 95% CI 3.71–9.11), pain (OR = 4.05, 95% CI 2.60–6.34), nausea and vomiting (OR = 3.01, 95% CI 1.81–5.02), and dyspnea (OR = 2.84, 95% CI 1.75–4.64), with less pronounced but still notable elevations for other symptoms. The data are shown in Figure 2 and are also provided in Supplementary Table S3a,b.

### 3.5. Gender-Stratified Multivariable Logistic Regression of PA, Nutritional and Sarcopenia Risk on Quality of Life

#### 3.5.1. Functional Scales and GHS by Gender

Functional scores generally increased across MET quartiles, with more consistent and pronounced improvements observed in men. Higher MET levels were associated with progressively higher odds of better scores in all domains, reaching the greatest increases in the Intensive group (physical OR = 6.90, 95% CI 3.48–13.97; role OR = 6.23, 95% CI 2.90–13.93; emotional OR = 3.65, 95% CI 1.89–7.11; cognitive OR = 3.70, 95% CI 1.80–7.81; social OR = 3.69, 95% CI 1.82–7.64; GHS OR = 4.10, 95% CI 2.12–8.01).

In women, improvements across MET quartiles were generally smaller and less consistent, with significant increases mainly observed for physical (OR = 3.40, 95% CI 1.62–7.23) and role functioning (OR = 4.76, 95% CI 2.17–10.62), while emotional, social, and GHS showed non-significant changes.

Both higher nutritional risk (NRS-2002 ≥ 3) and sarcopenia risk (SARC-F ≥ 4) were associated with reduced likelihood of higher functional scores in men and women. Men with SARC-F ≥ 4 had markedly lower odds of better scores in physical (OR = 0.17, 95% CI 0.09–0.33), role (OR = 0.15, 95% CI 0.08–0.28), and GHS (OR = 0.23, 95% CI 0.13–0.40), whereas reductions in women were smaller but significant for physical (OR = 0.13, 95% CI 0.06–0.27), emotional (OR = 0.29, 95% CI 0.14–0.59), and GHS (OR = 0.24, 95% CI 0.11–0.49). All details are shown in Supplementary Table S4a,b.

#### 3.5.2. Symptom Scales by Gender

Conversely, symptom scores generally decreased progressively across MET quartiles. MET levels were associated with lower odds of severe symptoms for men in all domains, with the largest reductions seen in the Intensive group (fatigue OR = 0.17, 95% CI 0.08–0.33; nausea and vomiting OR = 0.42, 95% CI 0.17–0.99; pain OR = 0.26, 95% CI 0.12–0.52; dyspnea OR = 0.24, 95% CI 0.09–0.57; insomnia OR = 0.28, 95% CI 0.13–0.55; appetite loss OR = 0.25, 95% CI 0.09–0.62; constipation OR = 0.31, 95% CI 0.15–0.66; diarrhea OR = 0.27, 95% CI 0.10–0.66; financial difficulties OR = 0.30, 95% CI 0.14–0.62).

In women, significant decreases were observed mainly in fatigue (OR = 0.34, 95% CI 0.16–0.73) and pain (OR = 0.38, 95% CI 0.18–0.80).

Nutritional risk (NRS-2002 ≥ 3) was associated with higher odds of severe symptoms in both genders, particularly fatigue (men OR = 3.13, 95% CI 1.79–5.48; women OR = 3.20, 95% CI 1.47–7.00) and appetite loss (men OR = 4.01, 95% CI 2.07–7.84; women OR = 5.95, 95% CI 2.51–14.47). Similarly, patients with higher sarcopenia risk (SARC-F ≥ 4) had higher odds of severe symptoms, especially fatigue (men OR = 5.60, 95% CI 3.13–10.07; women OR = 5.21, 95% CI 2.54–10.83), pain (men OR = 3.76, 95% CI 2.11–6.71; women OR = 4.35, 95% CI 2.12–8.97), and dyspnea (men OR = 3.15, 95% CI 1.67–5.98; women OR = 2.52, 95% CI 1.12–5.70). All data are shown in Supplementary Table S5a–c.

## 4. Discussion

This cross-sectional analysis provides valuable insights into PA levels in newly diagnosed cancer patients and their relationship with multiple dimensions of HRQoL, a key aspect in the early phase of the oncological pathway. In addition, nutritional and sarcopenia risk, as well as anthropometric and BC parameters, were collected to provide a more comprehensive characterization of patients’ clinical and functional status. It should be noted that, due to the cross-sectional design of this study, the observed relationships are associative and do not imply causality. For example, patients with better baseline functional status may be more likely to engage in PA, which could partially explain the associations observed between PA, nutritional status, and quality of life.

Accordingly, we observed that about 85.3% of patients presented a low nutritional risk (NRS-2002 < 3) and 83.7% were not at risk of sarcopenia (SARC-F < 4) at their first nutritional visit. Conversely, more than half of patients were overweight or obese (66%), which may partially reflect the relatively low nutritional risk observed in our cohort. These results about nutritional risk significantly differed from other studies. Notably, the PreMiO study, a prospective, multicenter study, showed 42.4% of cancer patients at nutritional risk and 8.7% malnourished at their first oncologic visit. Moreover, severity of malnutrition was positively correlated with the stage of cancer [51].

To better understand how nutritional status impacts on PA levels, our study also examined the potential influence of nutritional risk and BC on PA levels, through the assessment of muscle quantity and quality indices. In this context, malnutrition and sarcopenia risk decreased progressively while increasing PA, suggesting that higher activity levels may be associated with better nutritional and functional status. Similarly, in univariate analysis, patients who reported higher levels of PA demonstrated better BC parameters, as reflected by higher PhA. In this regard, Malveiro et al. [52] and Padilha et al. [53] reported that PA-based interventions, especially resistance exercises, can improve physical function, BC, and quality of life in cancer patients undergoing neoadjuvant and adjuvant therapies, specifically by increasing lean mass, muscle strength, and improving fat mass distribution. Likewise, the meta-analysis by Tian et al. (2022) found that engaging in PA, particularly resistance exercise, enhances BC parameters, such as lean mass, SMM, and physical function in patients with prostate cancer undergoing androgen deprivation therapy [54]. Overall, these findings emphasize the importance of implementing personalized PA interventions along cancer cure, in order to preserve nutritional status, BC and in particular muscle mass, whose deterioration has been associated with worse clinical outcomes, namely higher mortality risk, treatment discontinuation, postoperative complications and longer hospital stays [55,56].

In our study, we observed a clear and progressive relationship between PA levels, expressed as METs (MET-minutes/week), and quality of life in cancer patients. At univariate analysis, higher PA was associated with better scores across most functional domains, including physical, role, emotional, and social functioning, as well as GHS, whereas lower PA levels correlated with higher symptom burden, particularly fatigue, nausea and vomiting, pain, dyspnea, insomnia, and appetite loss. These associations were confirmed at multivariate analysis and remained statistically significant after adjustment for age, gender, cancer type, FFMI, and BCMI, except for cognitive functioning. Notably, the positive association between METs and quality of life increased progressively across MET categories, highlighting the potential benefits of high-intensity PA. Our results are consistent with current scientific evidence. In this regard, results from the cross-sectional analysis of Lopes et al. suggest that cancer survivors who met the World Health Organization’s recommendation of 150 min/week of PA had a better quality of life across phases of cancer survivorship, as well as reduced levels of cancer-related fatigue [57]. In line with these findings, a meta-synthesis of qualitative research indicates that PA positively influences several aspects of cancer patients’ quality of life, including physical, psychological, and social, regardless of diagnosis (i.e., stage, cancer type) and treatment status [58,59]. More specifically, Burke et al. [59] revealed that PA was related to improvements in overall physical fitness, energy levels, muscle strength, and sleep quality, as well as reductions in pain and physical discomfort. Moreover, PA may help mitigate adverse physical effects related to both the disease and its treatment, particularly fatigue and pain. Recent RCTs suggest that structured PA interventions, primarily including aerobic and resistance exercises, can improve quality of life and reduce anxiety in breast cancer survivors following the completion of anticancer therapies compared to usual care [60].

The beneficial effects of PA may be attributed to several mechanisms. First, it exerts anti-inflammatory effects by modulating inflammatory markers, such as tumor necrosis factor-α (TNF-α). Second, evidence indicates that physical exercise, particularly moderate-to-vigorous aerobic activity, can increase endogenous opioid levels, such as endorphins, thereby reducing pain and fatigue. Third, PA enhances the production of 5-hydroxytryptamine and dopamine, which in turn modulate reward and pleasure pathways, leading to reduced anxiety and improved quality of life [61].

In our analysis, lower quality of life scores in most functional scales, particularly in physical, role, and emotional domains, as well as in GHS, were reported in patients at risk of malnutrition, evaluated by the NRS-2002, compared to patients with adequate nutritional status. At the same time, patients at nutritional risk reported higher scores in symptom scales, particularly fatigue, nausea and vomiting, pain, dyspnea, insomnia, appetite loss and diarrhea. Similarly, patients at sarcopenia risk showed poorer functional scores across all domains, including physical, role, emotional, cognitive, social and GHS. Overall, these findings suggest that nutritional risk may be associated with poorer quality of life in cancer patients from early stages of diagnosis, contributing to reduced functional capacity and a greater symptom burden [62,63,64].

Nutritional status may also influence quality of life in cancer patients receiving palliative care, as observed in the large prospective cohort of de Oliveira et al. Patients with poorer nutritional status presented significantly lower scores in physical and emotional functioning scales, symptom domains, and overall quality of life. Moreover, patients with cancer-related cachexia reported higher symptom scores for dyspnea, insomnia, and appetite loss. Finally, the risk of malnutrition was significantly associated with impairment across all domains included in the Quality of Life Questionnaire—Core 15 Palliative [65], highlighting the need for structured assessment of nutritional status and quality of life in patients in advanced-stage cancers [66].

In our subgroup analyses, we observed potential gender-related differences in the association between nutritional risk, BC, PA levels, and HRQoL. Accordingly, male patients at risk of malnutrition were more frequently classified in the low PA category, whereas a similar trend was observed in women, although this result did not reach statistical significance. Conversely, women at higher risk of sarcopenia were more commonly represented among the least physically active participants. PhA increased significantly across higher PA levels in both genders. In men, additional significant increases were observed in ICW particularly among patients with higher activity, suggesting greater cellular and metabolically active mass in more physically active individuals. Analysis of HRQoL scores showed that, for both genders, selected functional scales (physical functioning and role functioning), Summary and Functional mean scores increased progressively from the Low to the Intensive group. In men, additional domains, including emotional, cognitive, social functioning, and GHS, also improved significantly across MET quartiles. In contrast, these associations were not observed in women. For symptomatic scales and cumulative score, we observed a significant decrease from the Low to Intensive group in men. Among women, significant reductions were observed only for fatigue and pain, while trends for nausea/vomiting and dyspnea did not reach statistical significance. These findings suggest that higher PA levels are associated with better BC and higher HRQoL, although the magnitude and pattern of these benefits may differ by gender. In this context, further studies are warranted to better characterize potential gender-specific differences in the association between PA levels and quality of life. They may also support the implementation of tailored nutritional and PA-based interventions to improve overall HRQoL and its specific domains.

This study has several strengths and limitations. First, the inclusion of a large cohort of newly diagnosed cancer patients across different cancer types enhances the robustness of our findings. Secondly, the systematic and comprehensive assessment of sociodemographic variables, lifestyle factors, nutritional status (with particular attention to malnutrition and sarcopenia risk), and BC evaluated by BIA, PA levels, and adherence to the MedDiet enabled a robust multifactorial analysis of HRQoL, accounting for key determinants identified in previous studies [32,67,68]. The subgroup analysis provided a more detailed evaluation of potential gender-specific differences in the relationships between nutritional risk, BC, PA, and HRQoL among cancer patients.

Regarding limitations, the single-center nature of the study may limit the generalizability of the findings, while the cross-sectional design did not allow us to establish causality. In addition, the present results are restricted to newly diagnosed cancer patients at the beginning of the diagnostic–therapeutic care pathway; therefore, no information is available on longitudinal changes in PA levels or on quality of life in relation to ongoing cancer treatments. Moreover, in this analysis, we used the SARC-F questionnaire as a screening tool to identify patients at risk of sarcopenia. However, objective assessments of MS—such as handgrip strength or the chair stand test, as recommended by the EWGSOP2 working group—were not performed. Consequently, we were unable to provide a comprehensive evaluation of patients with a confirmed diagnosis of sarcopenia within this population of cancer patients. In this sense, incorporating objective measures of MS would have strengthened the analysis, enabling a more comprehensive and detailed evaluation of the association between PA levels and quality of life in this patient population. Another important limitation is that the high prevalence of overweight and/or obese patients could mask the presence of individuals with sarcopenic obesity (SO). Since direct measures of MS were not available, we could not comprehensively evaluate the presence of SO in our sample. We therefore identified patients at risk using definitions developed jointly by the European Society for Clinical Nutrition and Metabolism (ESPEN) and the European Association for the Study of Obesity (EASO) [69]: BMI ≥ 30 kg/m^2^ and SARC-F score ≥ 4. Using this approach, only about 5% of participants were classified as at risk. An additional limitation of our study is the potential influence of gender-related differences in the perception and reporting of quality of life and PA levels. Men may tend to underreport symptoms and overestimate quality of life, whereas women may report lower quality of life and a higher perception of symptoms. Similarly, self-classification of PA intensity (moderate vs. intensive) may differ between sexes, potentially affecting the observed associations. A further limitation is the lack of information on the type of PA, which may differently affect BC and cellular health, potentially influencing the observed associations with ICW/TBW ratio [70,71,72,73]. Additionally, clinical variables—such as tumor stage, molecular subtype, histological grade, and patients’ performance status—were not included in the analysis. The absence of these tumor-specific characteristics represents a relevant limitation, as they may significantly influence the relationship between PA levels and quality of life in cancer patients, even at the beginning of their diagnostic and therapeutic path. Future studies should therefore incorporate these factors to comprehensively evaluate their potential impact on both PA levels and HRQoL. In this context, further prospective studies and lifestyle-based clinical trials are warranted to investigate the role of PA and its evolution over time in cancer patients, considering key determinants such as anticancer therapies and tumor characteristics, which may significantly influence both PA levels and quality of life.

## 5. Conclusions

In conclusion, this cross-sectional analysis suggests that higher levels of PA are associated with better HRQoL in newly diagnosed cancer patients, whereas lower activity levels, poor nutritional status, and risk of sarcopenia are linked to greater symptom burden and reduced functional outcomes. Our findings highlight associations between PA, nutritional status, and quality of life in cancer patients, underscoring the potential value of integrated assessment and individualized management strategies, without implying causal relationships or intervention effectiveness. Further randomized controlled trials are warranted to evaluate the effectiveness of such interventions and to clarify the optimal timing, intensity, duration, and type of PA required to enhance clinical outcomes and well-being in cancer patients.

## Figures and Tables

**Table 1:** Characteristics of the study cohort: sociodemographic, clinical, anthropometric, and quality of life variables.

Variable	N = 680 ^1^
Gender	
Female	290 (42.6%)
Male	390 (57.4%)
Age	61.8 (12.3)
Marital status	
Single	63 (9.5%)
Married or cohabiting	517 (77.7%)
Divorced or separated	45 (6.8%)
Widowed	40 (6.0%)
Education level	
Primary school	66 (10.0%)
Lower secondary school	199 (30%)
High school	253 (38.2%)
Graduation or post	144 (21.8%)
Smoking status	
Smoker	161 (24.4%)
Former smoker	285 (43.2%)
Non-smoker	214 (32.4%)
Cancer types	
Gastrointestinal	192 (28.2%)
Breast	82 (12.1%)
Melanoma	77 (11.3%)
Lung	99 (14.6%)
Head and neck	24 (3.5%)
Uro-gynecological	206 (30.3%)
Comorbidity	
No	273 (40.1%)
Yes	407 (59.9%)
NRS-2002	
<3	570 (85.3%)
≥3	98 (14.7%)
SARC-F	
<4	556 (83.7%)
≥4	108 (16.3%)
Weight (kg)	75.3 (16.3)
BMI (kg/m^2^)	
Normal weight	228 (34.1%)
Overweight	260 (39.0%)
Obese	180 (26.9%)
WC (cm)	96.2 (14.0)
HC (cm)	102.0 (11.4)
PhA (°)	5.6 (1.2)
ECW (L)	19.2 (4.2)
ICW (L)	20.8 (5.2)
BCMI (kg/m^2^)	10.1 (2.2)
FMI (kg/m^2^)	8.0 (3.8)
FFMI (kg/m^2^)	19.6 (2.6)
SMI (kg/m^2^)	9.2 (1.7)
ASMI (kg/m^2^)	7.4 (1.2)
MedDiet mean score (17 item)	7.7 (2.5)
<7	231 (34.0%)
≥7	449 (66.0%)
SumSc	80.0 (15.6)
Functional Score	78.7 (17.8)
Physical	81.5 (20.1)
Role	78.0 (29.3)
Emotional	69.4 (25.2)
Cognitive	85.1 (18.4)
Social	79.5 (24.2)
GHS	58.9 (24.3)
Symptom score	19.3 (15.4)
Fatigue	31.3 (25.7)
Nausea and vomiting	7.6 (16.0)
Pain	23.5 (26.4)
Dyspnea	16.7 (24.1)
Insomnia	31.2 (30.1)
Appetite loss	14.7 (25.5)
Constipation	20.5 (27.0)
Diarrhea	8.3 (19.0)
Financial difficulties	19.4 (26.9)
MET-minutes/week	
Low	125 (25.3%)
Moderate	123 (24.8%)
Moderate-to-High	123 (24.8%)
Intensive	124 (25.1%)

^1^ Mean (SD); n (%); NRS-2002, Nutritional Risk Screening 2002; SARC-F, Strength, Assistance with walking, Rise from a chair, Climb stairs, and Falls; BMI, Body Mass Index; WC, Waist circumference; HC, Hip circumference; PhA, Phase angle; ECW, Extracellular water; ICW, Intracellular water; BCMI, Body cell mass index; FMI, Fat mass index; FFMI, Fat-free mass index; SMI, Skeletal muscle mass index; ASMI, Appendicular skeletal muscle mass index; SumSc, Summary Score; GHS, Global Health Status; MedDiet, Mediterranean Diet; MET, Metabolic Equivalent.

**Table 2:** Associations between MET levels and participant characteristics.

Variable	Low N = 125 ^1^	Moderate N = 123 ^1^	Moderate to High N = 123 ^1^	Intensive N = 124 ^1^	*p*-Value ^2^
Gender	63.7 (12.5)	62.0 (11.4)	61.5 (13.6)	60.5 (10.8)	0.173
Female					0.374
Male	48 (38.4%)	48 (39.0%)	48 (39.0%)	61 (49.2%)	
Age	77 (61.6%)	75 (61.0%)	75 (61.0%)	63 (50.8%)	
Marital status					0.345
Single	11 (9.1%)	7 (5.9%)	18 (14.8%)	11 (8.9%)	
Married or cohabiting	99 (81.8%)	99 (83.2%)	86 (70.5%)	94 (76.4%)	
Divorced or separated	4 (3.3%)	6 (5.0%)	12 (9.8%)	9 (7.3%)	
Widowed	7 (5.8%)	7 (5.9%)	6 (4.9%)	9 (7.3%)	
Education level					0.272
Primary school	17 (14.2%)	14 (11.8%)	10 (8.2%)	10 (8.3%)	
Lower secondary school	41 (34.2%)	30 (25.2%)	29 (23.8%)	35 (28.9%)	
High school	46 (38.3%)	45 (37.8%)	48 (39.3%)	50 (41.3%)	
Graduation or post	16 (13.3%)	30 (25.2%)	35 (28.7%)	26 (21.5%)	
Smoking status					0.342
Smoker	30 (25.2%)	26 (22.0%)	30 (24.6%)	27 (22.0%)	
Former smoker	60 (50.4%)	56 (47.5%)	51 (41.8%)	46 (37.4%)	
Non-smoker	29 (24.4%)	36 (30.5%)	41 (33.6%)	50 (40.7%)	
Cancer types					**0.001**
Gastrointestinal	29 (23.2%)	52 (42.3%)	37 (30.1%)	27 (21.8%)	
Breast	10 (8.0%)	16 (13.0%)	12 (9.8%)	23 (18.5%)	
Melanoma	11 (8.8%)	8 (6.5%)	18 (14.6%)	16 (12.9%)	
Lung	23 (18.4%)	14 (11.4%)	16 (13.0%)	15 (12.1%)	
Head and neck	12 (9.6%)	4 (3.3%)	1 (0.8%)	3 (2.4%)	
Uro-gynecological	40 (32.0%)	29 (23.6%)	39 (31.7%)	40 (32.3%)	
Comorbidity					0.758
No	48 (38.4%)	55 (44.7%)	50 (40.7%)	47 (37.9%)	
Yes	77 (61.6%)	68 (55.3%)	73 (59.3%)	77 (62.1%)	
NRS-2002					**0.005**
<3	93 (76.9%)	103 (85.8%)	105 (86.1%)	117 (94.4%)	
≥3	28 (23.1%)	17 (14.2%)	17 (13.9%)	7 (5.6%)	
SARC-F					**<0.001**
<4	84 (70.0%)	103 (85.8%)	108 (88.5%)	113 (92.6%)	
≥4	36 (30.0%)	17 (14.2%)	14 (11.5%)	9 (7.4%)	
Weight (kg)	75.7 (18.4)	75.1 (16.2)	74.7 (15.2)	74.8 (15.2)	0.983
BMI (kg/m^2^)					0.826
Normal weight	40 (33.1%)	41 (34.2%)	48 (39.3%)	39 (31.5%)	
Overweight	45 (37.2%)	50 (41.7%)	46 (37.7%)	50 (40.3%)	
Obese	36 (29.8%)	29 (24.2%)	28 (23.0%)	35 (28.2%)	
WC (cm)	98.5 (14.8)	94.8 (14.0)	95.8 (13.3)	94.8 (13.1)	0.712
HC (cm)	103.1 (12.2)	100.3 (11.8)	101.3 (10.7)	102.4 (10.5)	0.510
PhA (°)	5.2 (1.3)	5.7 (0.9)	5.6 (1.3)	6.0 (1.2)	**<0.001**
ECW (L)	19.9 (4.2)	19.2 (4.2)	19.4 (4.5)	18.5 (4.0)	0.107
ICW (L)	19.8 (5.8)	21.4 (4.7)	20.7 (5.2)	21.6 (5.5)	0.073
BCMI (kg/m^2^)	9.6 (2.5)	10.3 (1.8)	9.9 (2.1)	10.6 (2.0)	**0.006**
FMI (kg/m^2^)	8.6 (4.4)	7.5 (3.6)	7.7 (3.5)	7.6 (3.3)	0.385
FFMI (kg/m^2^)	19.6 (2.9)	19.8 (2.5)	19.4 (2.5)	19.9 (2.5)	0.712
SMI (kg/m^2^)	9.2 (1.8)	9.3 (1.6)	9.1 (1.6)	9.2 (1.7)	0.826
ASMI (kg/m^2^)	7.4 (1.4)	7.5 (1.1)	7.3 (1.2)	7.6 (1.2)	0.684
MedDiet Score (%)					0.919
<7	47 (37.6%)	43 (35.0%)	44 (35.8%)	41 (33.1%)	
≥7	78 (62.4%)	80 (65.0%)	79 (64.2%)	83 (66.9%)	
SumSc	71.6 (18.6)	79.2 (13.8)	83.6 (13.3)	85.7 (12.3)	**<0.001**
Functional Score	69.4 (20.3)	77.6 (16.6)	83.4 (14.6)	84.6 (14.6)	**<0.001**
Physical	68.8 (25.4)	83.5 (15.7)	86.6 (15.4)	89.9 (12.0)	**<0.001**
Role	61.9 (36.6)	77.4 (26.2)	84.3 (23.9)	88.6 (19.3)	**<0.001**
Emotional	63.7 (27.6)	65.9 (26.0)	74.1 (22.0)	74.5 (23.8)	**0.003**
Cognitive	81.9 (19.6)	84.1 (18.8)	88.2 (17.0)	86.7 (19.2)	0.052
Social	70.8 (29.8)	77.1 (24.5)	83.9 (21.0)	83.5 (21.7)	**0.001**
GHS	49.5 (26.7)	56.6 (23.2)	63.0 (20.1)	65.4 (23.4)	**<0.001**
Symptom Score	26.8 (18.2)	19.7 (14.0)	16.1 (14.0)	13.9 (12.2)	**<0.001**
Fatigue	44.9 (28.6)	31.4 (23.7)	25.7 (22.5)	22.6 (21.4)	**<0.001**
Nausea and vomiting	13.6 (22.6)	6.8 (14.4)	5.8 (11.9)	4.4 (9.5)	**0.008**
Pain	35.3 (31.4)	25.2 (25.3)	18.2 (22.1)	16.4 (21.1)	**<0.001**
Dyspnea	23.2 (28.5)	17.6 (23.1)	15.2 (22.7)	8.6 (16.4)	**<0.001**
Insomnia	38.7 (32.6)	32.2 (29.5)	27.4 (29.9)	27.4 (27.9)	**0.029**
Appetite loss	23.5 (31.4)	14.4 (24.5)	11.4 (22.5)	9.7 (20.7)	**0.001**
Constipation	25.9 (29.6)	21.1 (28.1)	19.2 (26.7)	15.3 (21.4)	0.080
Diarrhea	11.5 (22.4)	9.8 (22.9)	7.3 (17.4)	5.1 (14.1)	0.173
Financial difficulties	24.3 (30.3)	18.7 (27.4)	15.2 (24.6)	15.3 (20.6)	0.107

^1^ Mean (SD), n (%); ^2^ Kruskal–Wallis rank sum test, Pearson’s Chi-squared test; NRS-2002, Nutritional Risk Screening 2002; SARC-F, Strength, Assistance with walking, Rise from a chair, Climb stairs, and Falls; BMI, Body Mass Index; WC, Waist circumference; HC, Hip circumference; PhA, Phase angle; ECW, Extracellular water; ICW, Intracellular water; BCMI, Body cell mass index; FMI, Fat mass index; FFMI, Fat-free mass index; SMI, Skeletal muscle mass index; ASMI, Appendicular Skeletal muscle mass index; SumSc, Summary Score; GHS, Global Health Status; MedDiet, Mediterranean Diet.

## Data Availability

The original data presented in this study are openly available from Zenodo at https://doi.org/10.5281/zenodo.18347187.

## Supplementary Materials

The following supporting information can be downloaded at: https://www.mdpi.com/article/10.3390/nu18050844/s1, Table S1: Associations between MET Levels and Participant Characteristics by Gender; Table S2: Multivariable-adjusted models for the association of physical activity levels with functional HRQoL scales; Table S3a: Multivariable-adjusted models for the association of physical activity levels with symptom HRQoL scales; Table S3b: Multivariable-adjusted models for the association of physical activity levels with symptom HRQoL scales; Table S4a: Multivariable-adjusted models for the association of physical activity levels with symptom HRQoL scales stratified by gender; Table S4b: Multivariable-adjusted models for the association of physical activity levels with functional HRQoL scales stratified by gender; Table S5a: Multivariable-adjusted models for the association of physical activity levels with symptoms HRQoL scales stratified by gender; Table S5b: Multivariable-adjusted models for the association of physical activity levels with symptoms HRQoL scales stratified by gender. Table S5c: Multivariable-adjusted models for the association of physical activity levels with symptoms HRQoL scales stratified by gender.

## Abbreviations

The following abbreviations are used in this manuscript:

ASMI	Appendicular Skeletal Muscle Index
ASMM	Appendicular Skeletal Muscle Mass
BC	Body Composition
BCM	Body Cell Mass
BCMI	Body Cell Mass Index
BIA	Bioelectrical Impedance Analysis
BMI	Body Mass Index
CRF	Case Report Form
ECW	Extracellular Water
FDR	False Discovery Rate
FFM	Fat-Free Mass
FFMI	Fat-Free Mass Index
FM	Fat Mass
FMI	Fat Mass Index
GHS	Global Health Status
HC	Hip Circumference
HRQoL	Health-Related Quality Of Life
ICIs	Immune Checkpoint Inhibitors
ICW	Intracellular Water
IPAQ-SF	International Physical Activity Questionnaire–Short Form
MedDiet	Mediterranean Diet
MET	Metabolic Equivalent
MOGs	Multidisciplinary Oncology Groups
MS	Muscle Strength
NRS-2002	Nutritional Risk Screening 2002
PA	Physical Activity
PhA	Phase Angle
PN	Peripheral Neuropathy
SARC-F	Strength, Assistance With Walking, Rise From A Chair, Climb Stairs, And Falls
SD	Standard Deviation
SMI	Skeletal Muscle Index
SMM	Skeletal Muscle Mass
SO	Sarcopenic Obesity
SumSc	C30 Summary Score
WC	Waist Circumference
WHO	World Health Organization

## Appendix A

The NUTRISCREEN research group includes Michelino De Laurentiis, Francesco Auriemma, Ludovica Orsi, Adele Tatarella (Department of Breast and Thoracic Oncology, Istituto Nazionale Tumori, IRCCS-Fondazione G. Pascale, Naples, Italy); Francesco Perri, Vincenza Liccardo, Claudia Olivetta (Medical and Experimental Head and Neck Oncology Unit, Istituto Nazionale Tumori, IRCCS-Fondazione G. Pascale, Naples, Italy); Francesca Foschini, Lucrezia Silvestro, Anna Nappi, Antonio Daniele, Carmela Romano, Pasqualina Perrotta (Experimental Clinical Abdominal Oncology Unit, Istituto Nazionale Tumori IRCCS-Fondazione G. Pascale, Naples, Italy); Alessandro Morabito, Simona Damiano, Amalia Illiano (Thoracic Medical Oncology, Istituto Nazionale Tumori IRCCS-Fondazione G. Pascale, Naples, Italy); Paolo Antonio Ascierto, Assunta Calignano (Melanoma Cancer Immunotherapy and Innovative Therapy Unit, Istituto Nazionale Tumori IRCCS-Fondazione G. Pascale, Naples, Italy); Rosa Tambaro, Sabrina Rossetti, Marilena Di Napoli, Carmela Pisano, Sabrina Chiara Cecere, Giuseppina Canciello, Gelsomina Iovane, Rossella Noce, Lucia Formisano, and Rosaria Grimaldi (Department of Urology and Gynecology, Istituto Nazionale Tumori IRCCS-Fondazione G. Pascale, Naples, Italy).

## References

[1] Bray F., Laversanne M., Sung H., Ferlay J., Siegel R.L., Soerjomataram I., Jemal A. (2024). Global Cancer Statistics 2022: GLOBOCAN Estimates of Incidence and Mortality Worldwide for 36 Cancers in 185 Countries. CA. Cancer J. Clin..

[2] Marino P., Mininni M., Deiana G., Marino G., Divella R., Bochicchio I., Giuliano A., Lapadula S., Lettini A.R., Sanseverino F. (2024). Healthy Lifestyle and Cancer Risk: Modifiable Risk Factors to Prevent Cancer. Nutrients.

[3] The National Cancer Institute Physical Activity and Cancer. https://www.cancer.gov/about-cancer/causes-prevention/risk/obesity/physical-activity-fact-sheet.

[4] Dieli-Conwright C.M., Courneya K.S., Demark-Wahnefried W., Sami N., Lee K., Sweeney F.C., Stewart C., Buchanan T.A., Spicer D., Tripathy D. (2018). Aerobic and Resistance Exercise Improves Physical Fitness, Bone Health, and Quality of Life in Overweight and Obese Breast Cancer Survivors: A Randomized Controlled Trial. Breast Cancer Res..

[5] Samuel S.R., Maiya A.G., Fernandes D.J., Guddattu V., Saxena P.P., Kurian J.R., Lin P.-J., Mustian K.M. (2019). Effectiveness of Exercise-Based Rehabilitation on Functional Capacity and Quality of Life in Head and Neck Cancer Patients Receiving Chemo-Radiotherapy. Support. Care Cancer.

[6] Rodríguez-Cañamero S., Cobo-Cuenca A.I., Carmona-Torres J.M., Pozuelo-Carrascosa D.P., Santacruz-Salas E., Rabanales-Sotos J.A., Cuesta-Mateos T., Laredo-Aguilera J.A. (2022). Impact of Physical Exercise in Advanced-stage Cancer Patients: Systematic Review and Meta-analysis. Cancer Med..

[7] Aydin M., Kose E., Odabas I., Meric Bingul B., Demirci D., Aydin Z. (2021). The Effect of Exercise on Life Quality and Depression Levels of Breast Cancer Patients. Asian Pac. J. Cancer Prev..

[8] Rendeiro J.A., Rodrigues C.A.M.P., De Barros Rocha L., Rocha R.S.B., Da Silva M.L., Da Costa Cunha K. (2021). Physical Exercise and Quality of Life in Patients with Prostate Cancer: Systematic Review and Meta-Analysis. Support. Care Cancer.

[9] Stamatakis E., Ahmadi M.N., Gill J.M.R., Thøgersen-Ntoumani C., Gibala M.J., Doherty A., Hamer M. (2022). Association of Wearable Device-Measured Vigorous Intermittent Lifestyle Physical Activity with Mortality. Nat. Med..

[10] Moore S.C., Lee I.-M., Weiderpass E., Campbell P.T., Sampson J.N., Kitahara C.M., Keadle S.K., Arem H., Berrington De Gonzalez A., Hartge P. (2016). Association of Leisure-Time Physical Activity With Risk of 26 Types of Cancer in 1.44 Million Adults. JAMA Intern. Med..

[11] Friedenreich C.M., Stone C.R., Cheung W.Y., Hayes S.C. (2020). Physical Activity and Mortality in Cancer Survivors: A Systematic Review and Meta-Analysis. JNCI Cancer Spectr..

[12] Mctiernan A., Friedenreich C.M., Katzmarzyk P.T., Powell K.E., Macko R., Buchner D., Pescatello L.S., Bloodgood B., Tennant B., Vaux-Bjerke A. (2019). Physical Activity in Cancer Prevention and Survival: A Systematic Review. Med. Sci. Sports Exerc..

[13] Friedenreich C.M., Neilson H.K., Lynch B.M. (2010). State of the Epidemiological Evidence on Physical Activity and Cancer Prevention. Eur. J. Cancer.

[14] Zhou Y., Jia N., Ding M., Yuan K. (2022). Effects of Exercise on Inflammatory Factors and IGF System in Breast Cancer Survivors: A Meta-Analysis. BMC Womens Health.

[15] Khandekar M.J., Cohen P., Spiegelman B.M. (2011). Molecular Mechanisms of Cancer Development in Obesity. Nat. Rev. Cancer.

[16] Santa Mina D., Clarke H., Ritvo P., Leung Y.W., Matthew A.G., Katz J., Trachtenberg J., Alibhai S.M.H. (2014). Effect of Total-Body Prehabilitation on Postoperative Outcomes: A Systematic Review and Meta-Analysis. Physiotherapy.

[17] Barberan-Garcia A., Ubré M., Roca J., Lacy A.M., Burgos F., Risco R., Momblán D., Balust J., Blanco I., Martínez-Pallí G. (2018). Personalised Prehabilitation in High-Risk Patients Undergoing Elective Major Abdominal Surgery: A Randomized Blinded Controlled Trial. Ann. Surg..

[18] Van Vulpen J.K., Peeters P.H.M., Velthuis M.J., Van Der Wall E., May A.M. (2016). Effects of Physical Exercise during Adjuvant Breast Cancer Treatment on Physical and Psychosocial Dimensions of Cancer-Related Fatigue: A Meta-Analysis. Maturitas.

[19] Schumacher O., Galvão D.A., Taaffe D.R., Chee R., Spry N., Newton R.U. (2021). Exercise Modulation of Tumour Perfusion and Hypoxia to Improve Radiotherapy Response in Prostate Cancer. Prostate Cancer Prostatic Dis..

[20] Liu J., Liu W., Wan Y., Mao W. (2024). Crosstalk between Exercise and Immunotherapy: Current Understanding and Future Directions. Research.

[21] Del Bianco N., Borsati A., Toniolo L., Ciurnielli C., Belluomini L., Insolda J., Sposito M., Milella M., Schena F., Pilotto S. (2024). What Is the Role of Physical Exercise in the Era of Cancer Prehabilitation? A Systematic Review. Crit. Rev. Oncol. Hematol..

[22] Voorn M.J.J., Driessen E.J.M., Reinders R.J.E.F., Van Kampen-van Den Boogaart V.E.M., Bongers B.C., Janssen-Heijnen M.L.G. (2023). Effects of Exercise Prehabilitation and/or Rehabilitation on Health-Related Quality of Life and Fatigue in Patients with Non-Small Cell Lung Cancer Undergoing Surgery: A Systematic Review. Eur. J. Surg. Oncol..

[23] Avancini A., Borsati A., Toniolo L., Ciurnelli C., Belluomini L., Budolfsen T., Lillelund C., Milella M., Quist M., Pilotto S. (2025). Physical Activity Guidelines in Oncology: A Systematic Review of the Current Recommendations. Crit. Rev. Oncol. Hematol..

[24] Gil F., Costa G., Hilker I., Benito L. (2012). First Anxiety, Afterwards Depression: Psychological Distress in Cancer Patients at Diagnosis and after Medical Treatment. Stress Health.

[25] Weaver K.E., Forsythe L.P., Reeve B.B., Alfano C.M., Rodriguez J.L., Sabatino S.A., Hawkins N.A., Rowland J.H. (2012). Mental and Physical Health–Related Quality of Life among U.S. Cancer Survivors: Population Estimates from the 2010 National Health Interview Survey. Cancer Epidemiol. Biomark. Prev..

[26] The WHOQOL Group (1995). The World Health Organization Quality of Life Assessment (WHOQOL): Position Paper from the World Health Organization. Soc. Sci. Med..

[27] Mierzynska J., Piccinin C., Pe M., Martinelli F., Gotay C., Coens C., Mauer M., Eggermont A., Groenvold M., Bjordal K. (2019). Prognostic Value of Patient-Reported Outcomes from International Randomised Clinical Trials on Cancer: A Systematic Review. Lancet Oncol..

[28] Segal R., Zwaal C., Green E., Tomasone J.R., Loblaw A., Petrella T., The Exercise for People with Cancer Guideline Development Group (2017). Exercise for People with Cancer: A Systematic Review. Curr. Oncol..

[29] Stout N.L., Santa Mina D., Lyons K.D., Robb K., Silver J.K. (2021). A Systematic Review of Rehabilitation and Exercise Recommendations in Oncology Guidelines. CA. Cancer J. Clin..

[30] Zhang X., Tang M., Zhang Q., Zhang K.-P., Guo Z.-Q., Xu H.-X., Yuan K.-T., Yu M., Braga M., Cederholm T. (2021). The GLIM Criteria as an Effective Tool for Nutrition Assessment and Survival Prediction in Older Adult Cancer Patients. Clin. Nutr..

[31] Arends J., Baracos V., Bertz H., Bozzetti F., Calder P.C., Deutz N.E.P., Erickson N., Laviano A., Lisanti M.P., Lobo D.N. (2017). ESPEN Expert Group Recommendations for Action against Cancer-Related Malnutrition. Clin. Nutr..

[32] Porciello G., Di Lauro T., Luongo A., Coluccia S., Prete M., Abbadessa L., Coppola E., Di Martino A., Mozzillo A.L., Racca E. (2025). Optimizing Nutritional Care with Machine Learning: Identifying Sarcopenia Risk Through Body Composition Parameters in Cancer Patients—Insights from the NUTritional and Sarcopenia RIsk SCREENing Project (NUTRISCREEN). Nutrients.

[33] Wang L., Dai X., Ai J., Li X., Deng A., Yang X., Zhang Y., Tian D., He L. (2025). The Relationship between Sarcopenia, Nutritional Status, Physical Function, and Quality of Life in Elderly Cancer Patients: A Path Analysis. BMC Geriatr..

[34] Cruz-Jentoft A.J., Bahat G., Bauer J., Boirie Y., Bruyère O., Cederholm T., Cooper C., Landi F., Rolland Y., Sayer A.A. (2019). Sarcopenia: Revised European Consensus on Definition and Diagnosis. Age Ageing.

[35] Looby M., Matthews L., West C.T., Khan K., Ansell G., Donovan K., Wood L., Tapley P., Lewis R., Stoddard K. (2025). Physical Fitness and Body Composition Assessments in Advanced Cancer Patients Undergoing Exenterative Surgery—A Pilot Cohort Study. Colorectal Dis..

[36] Cho E., Chodzko M., Compton S.L.E., Yang S., Heymsfield S., Spielmann G., Brown J.C. (2025). Effects of Aerobic Exercise on Body Composition and Exerkines in Colorectal Cancer Survivors. Front. Sports Act. Living.

[37] Lee J., Hwang Y. (2025). The Effects of Exercise Interventions on Fatigue, Body Composition, Physical Fitness, and Biomarkers in Breast Cancer Patients during and after Treatment: A Systematic Review and Meta-Analysis of Randomized Controlled Trials. J. Cancer Surviv..

[38] Courneya K.S., McNeely M.L., Booth C.M., Friedenreich C.M. (2024). An Integrated Framework for the Study of Exercise across the Postdiagnosis Cancer Continuum. Front. Oncol..

[39] Kondrup J., Rasmussen H.H., Hamberg O., Stanga Z., Ad Hoc ESPEN Working Group (2003). Nutritional Risk Screening (NRS 2002): A New Method Based on an Analysis of Controlled Clinical Trials. Clin. Nutr. Edinb. Scotl..

[40] Craig C.L., Marshall A.L., Sjöström M., Bauman A.E., Booth M.L., Ainsworth B.E., Pratt M., Ekelund U., Yngve A., Sallis J.F. (2003). International Physical Activity Questionnaire: 12-Country Reliability and Validity. Med. Sci. Sports Exerc..

[41] Lee P.H., Macfarlane D.J., Lam T.H., Stewart S.M. (2011). Validity of the International Physical Activity Questionnaire Short Form (IPAQ-SF): A Systematic Review. Int. J. Behav. Nutr. Phys. Act..

[42] van Poppel M.N.M., Chinapaw M.J.M., Mokkink L.B., van Mechelen W., Terwee C.B. (2010). Physical Activity Questionnaires for Adults: A Systematic Review of Measurement Properties. Sports Med. Auckl. NZ.

[43] Jetté M., Sidney K., Blümchen G. (1990). Metabolic Equivalents (METS) in Exercise Testing, Exercise Prescription, and Evaluation of Functional Capacity. Clin. Cardiol..

[44] Clinton S.K., Giovannucci E.L., Hursting S.D. (2020). The World Cancer Research Fund/American Institute for Cancer Research Third Expert Report on Diet, Nutrition, Physical Activity, and Cancer: Impact and Future Directions. J. Nutr..

[45] Aaronson N.K., Ahmedzai S., Bergman B., Bullinger M., Cull A., Duez N.J., Filiberti A., Flechtner H., Fleishman S.B., de Haes J.C. (1993). The European Organization for Research and Treatment of Cancer QLQ-C30: A Quality-of-Life Instrument for Use in International Clinical Trials in Oncology. J. Natl. Cancer Inst..

[46] Hinz A., Einenkel J., Briest S., Stolzenburg J.-U., Papsdorf K., Singer S. (2012). Is It Useful to Calculate Sum Scores of the Quality of Life Questionnaire EORTC QLQ-C30?: Sum Scores of EORTC QLQ-C30. Eur. J. Cancer Care.

[47] Schröder H., Zomeño M.D., Martínez-González M.A., Salas-Salvadó J., Corella D., Vioque J., Romaguera D., Martínez J.A., Tinahones F.J., Miranda J.L. (2021). Validity of the Energy-Restricted Mediterranean Diet Adherence Screener. Clin. Nutr. Edinb. Scotl..

[48] Galilea-Zabalza I., Buil-Cosiales P., Salas-Salvadó J., Toledo E., Ortega-Azorín C., Díez-Espino J., Vázquez-Ruiz Z., Zomeño M.D., Vioque J., Martínez J.A. (2018). Mediterranean Diet and Quality of Life: Baseline Cross-Sectional Analysis of the PREDIMED-PLUS Trial. PLoS ONE.

[49] Malcomson F.C., Wiggins C., Parra-Soto S., Ho F.K., Celis-Morales C., Sharp L., Mathers J.C. (2023). Adherence to the 2018 World Cancer Research Fund/American Institute for Cancer Research Cancer Prevention Recommendations and Cancer Risk: A Systematic Review and Meta-Analysis. Cancer.

[50] Sjostrom M., Ainsworth B.E., Bauman A., Bull F.C., Hamilton-Craig C.R., Sallis J.F. Guidelines for data processing analysis of the International Physical Activity Questionnaire (IPAQ)—Short and long forms. https://api.semanticscholar.org/CorpusID:79242415.

[51] Muscaritoli M., Lucia S., Farcomeni A., Lorusso V., Saracino V., Barone C., Plastino F., Gori S., Magarotto R., Carteni G. (2017). Prevalence of Malnutrition in Patients at First Medical Oncology Visit: The PreMiO Study. Oncotarget.

[52] Malveiro C., Correia I.R., Cargaleiro C., Magalhães J.P., De Matos L.V., Hilário S., Sardinha L.B., Cardoso M.J. (2023). Effects of Exercise Training on Cancer Patients Undergoing Neoadjuvant Treatment: A Systematic Review. J. Sci. Med. Sport.

[53] Padilha C.S., Marinello P.C., Galvão D.A., Newton R.U., Borges F.H., Frajacomo F., Deminice R. (2017). Evaluation of Resistance Training to Improve Muscular Strength and Body Composition in Cancer Patients Undergoing Neoadjuvant and Adjuvant Therapy: A Meta-Analysis. J. Cancer Surviv..

[54] Tian S., Ding M., Sun H. (2022). The Effects of Resistance Exercise on Body Composition and Physical Function in Prostate Cancer Patients Undergoing Androgen Deprivation Therapy: An Update Systematic Review and Meta-Analysis. Aging Male.

[55] Onishi S., Tajika M., Tanaka T., Yamada K., Kamiya T., Abe T., Higaki E., Fujieda H., Nagao T., Inaba Y. (2022). Effect of Body Composition Change during Neoadjuvant Chemotherapy for Esophageal Squamous Cell Carcinoma. J. Clin. Med..

[56] Molfino A., Imbimbo G., Pisegna S., Scagnoli S., Alabiso C., Ardovino M., Gallicchio C., Rizzo V., Botticelli A. (2025). Impact of Body Composition Changes on Treatment-Related Toxicities and Clinical Outcomes in HER2-Positive Metastatic Breast Cancer Patients Receiving Trastuzumab Deruxtecan. Cancers.

[57] Lopes M., Souza Cassaroti V.S., Cella P.S., Palma L.P., Dalcin V.S., de Moura M.O., Prianti D.E., Borges M.B.D.S., Godinho L.C., Terziotti F. (2025). Physical Activity Is Differently Associated with Quality of Life and Fatigue Across the Phases of Cancer Survivorship. Int. J. Sports Med..

[58] Dinas P.C., Karaventza M., Liakou C., Georgakouli K., Bogdanos D., Metsios G.S., On Behalf Of The Students Of Module (Introduction To Systematic Reviews) (2024). Combined Effects of Physical Activity and Diet on Cancer Patients: A Systematic Review and Meta-Analysis. Nutrients.

[59] Burke S., Wurz A., Bradshaw A., Saunders S., West M.A., Brunet J. (2017). Physical Activity and Quality of Life in Cancer Survivors: A Meta-Synthesis of Qualitative Research. Cancers.

[60] Sun M., Liu C., Lu Y., Zhu F., Li H., Lu Q. (2023). Effects of Physical Activity on Quality of Life, Anxiety and Depression in Breast Cancer Survivors: A Systematic Review and Meta-Analysis. Asian Nurs. Res..

[61] Sun Y., Ma Y., Shi L., Liu T., Dong Y., Jin Q. (2025). The Impact and Molecular Mechanisms of Exercise in Cancer Therapy. Curr. Issues Mol. Biol..

[62] Polański J., Jankowska-Polańska B., Mazur G. (2021). Relationship Between Nutritional Status and Quality of Life in Patients with Lung Cancer. Cancer Manag. Res..

[63] Maia F.D.C.P., Silva T.A., Generoso S.D.V., Correia M.I.T.D. (2020). Malnutrition Is Associated with Poor Health-Related Quality of Life in Surgical Patients with Gastrointestinal Cancer. Nutrition.

[64] Yu K., Zhou X., He S. (2013). A Multicentre Study to Implement Nutritional Risk Screening and Evaluate Clinical Outcome and Quality of Life in Patients with Cancer. Eur. J. Clin. Nutr..

[65] Groenvold M., Petersen M.A., Aaronson N.K., Arraras J.I., Blazeby J.M., Bottomley A., Fayers P.M., de Graeff A., Hammerlid E., Kaasa S. (2006). The Development of the EORTC QLQ-C15-PAL: A Shortened Questionnaire for Cancer Patients in Palliative Care. Eur. J. Cancer.

[66] De Oliveira L.C., Abreu G.T., Lima L.C., Aredes M.A., Wiegert E.V.M. (2020). Quality of Life and Its Relation with Nutritional Status in Patients with Incurable Cancer in Palliative Care. Support. Care Cancer.

[67] Xia X., Cao X., Gong C., Liu Y., Zhang X., Liao L. (2025). Adherence to the Mediterranean Diet Is Associated with Lower Cancer-Related Fatigue: A Cross-Sectional Analysis from NHANES 2017–2020. Front. Nutr..

[68] Porciello G., Montagnese C., Crispo A., Grimaldi M., Libra M., Vitale S., Palumbo E., Pica R., Calabrese I., Cubisino S. (2020). Mediterranean Diet and Quality of Life in Women Treated for Breast Cancer: A Baseline Analysis of DEDiCa Multicentre Trial. PLoS ONE.

[69] Donini L.M., Busetto L., Bischoff S.C., Cederholm T., Ballesteros-Pomar M.D., Batsis J.A., Bauer J.M., Boirie Y., Cruz-Jentoft A.J., Dicker D. (2022). Definition and Diagnostic Criteria for Sarcopenic Obesity: ESPEN and EASO Consensus Statement. Obes. Facts.

[70] Da Rocha N.S., Schuch F.B., De Almeida Fleck M.P. (2014). Gender Differences in Perception of Quality of Life in Adults with and without Chronic Health Conditions: The Role of Depressive Symptoms. J. Health Psychol..

[71] Skevington S.M., Schick-Makaroff K., Rowland C., Molzahn A., the WHOQOL Group (2024). Women’s Environmental Quality of Life Is Key to Their Overall Quality of Life and Health: Global Evidence from the WHOQOL-100. PLoS ONE.

[72] Oliveira A.S., Lopes S., Ferreira L.N., Cruz V.T., Costa A.R. (2025). Sex Differences in Health-Related Quality of Life among Individuals at High Risk of Dementia. Eur. Geriatr. Med..

[73] Cherepanov D., Palta M., Fryback D.G., Robert S.A. (2010). Gender Differences in Health-Related Quality-of-Life Are Partly Explained by Sociodemographic and Socioeconomic Variation between Adult Men and Women in the US: Evidence from Four US Nationally Representative Data Sets. Qual. Life Res..

