# Supplementary material for: Physical Activity, Nutritional Status, and Health-Related Quality of Life in Newly Diagnosed Cancer Patients: Evidence from the NUTRISCREEN Project
Source: Nutrients. 2026 Mar 5;18(5):844. doi: 10.3390/nu18050844 (PMC12986902; doi:10.3390/nu18050844)
Supplement: Supplementary file 1 [file nutrients-18-00844-s001.zip › nutrients-4160846-supplementary.pdf]

SUPPLEMENTARY MATERIAL

Supplementary Table S1
Supplementary Table S2
Supplementary Table S3a
Supplementary Table S3b
Supplementary Table S4a
Supplementary Table S4b
Supplementary Table S5a
Supplementary Table S5b
Supplementary Table S5c

Table S1 Associations between MET Levels and Participant Characteristics by Gender

Variable	Low<br>N = 48[1]	Moderate<br>N = 48[1]	Moderate to High<br>N = 48[1]	Intensive<br>N = 61[1]	p-value[2]	Low<br>N = 77[1]	Moderate<br>N = 75[1]	Moderate to High<br>N = 75[1]	Intensive<br>N = 63[1]	p-value[2]
	**Female**					**Male**				
**Age**	60.2 (16.2)	56.9 (12.7)	54.3 (13.7)	56.8 (10.6)	0.317	65.9 (9.0)	65.3 (9.2)	66.2 (11.5)	64.1 (9.8)	0.625
**Marital status**					0.796					0.360
Single	6 (12.8%)	5 (10.9%)	11 (22.9%)	6 (10.0%)		5 (6.8%)	2 (2.7%)	7 (9.5%)	5 (7.9%)	
Married or cohabiting	33 (70.2%)	34 (73.9%)	29 (60.4%)	41 (68.3%)		66 (89.2%)	65 (89.0%)	57 (77.0%)	53 (84.1%)	
Divorced or separated	3 (6.4%)	2 (4.3%)	4 (8.3%)	7 (11.7%)		1 (1.4%)	4 (5.5%)	8 (10.8%)	2 (3.2%)	
Widowed	5 (10.6%)	5 (10.9%)	4 (8.3%)	6 (10.0%)		2 (2.7%)	2 (2.7%)	2 (2.7%)	3 (4.8%)	
**Education level**					0.344					0.350
Primary school	9 (19.1%)	8 (17.4%)	2 (4.2%)	4 (6.8%)		8 (11.0%)	6 (8.2%)	8 (10.8%)	6 (9.7%)	
Lower secondary school	10 (21.3%)	9 (19.6%)	10 (20.8%)	9 (15.3%)		31 (42.5%)	21 (28.8%)	19 (25.7%)	26 (41.9%)	

High school	19 (40.4%)	14 (30.4%)	19 (39.6%)	28 (47.5%)		27 (37.0%)	31 (42.5%)	29 (39.2%)	22 (35.5%)	
Graduation or post	9 (19.1%)	15 (32.6%)	17 (35.4%)	18 (30.5%)		7 (9.6%)	15 (20.5%)	18 (24.3%)	8 (12.9%)	
**Smoking status**					0.878					0.096
Smoker	8 (17.4%)	10 (21.7%)	12 (25.0%)	14 (23.3%)		22 (30.1%)	16 (22.2%)	18 (24.3%)	13 (20.6%)	
Former smoker	15 (32.6%)	17 (37.0%)	12 (25.0%)	16 (26.7%)		45 (61.6%)	39 (54.2%)	39 (52.7%)	30 (47.6%)	
Non-smoker	23 (50.0%)	19 (41.3%)	24 (50.0%)	30 (50.0%)		6 (8.2%)	17 (23.6%)	17 (23.0%)	20 (31.7%)	
**Cancer types**					0.143					**0.065**
Gastrointestinal	9 (18.8%)	18 (37.5%)	9 (18.8%)	10 (16.4%)		20 (26.0%)	34 (45.3%)	28 (37.3%)	17 (27.0%)	
Breast	10 (20.8%)	16 (33.3%)	12 (25.0%)	23 (37.7%)		0 (0.0%)	0 (0.0%)	0 (0.0%)	0 (0.0%)	
Melanoma	6 (12.5%)	2 (4.2%)	10 (20.8%)	8 (13.1%)		5 (6.5%)	6 (8.0%)	8 (10.7%)	8 (12.7%)	
Lung	9 (18.8%)	4 (8.3%)	3 (6.3%)	5 (8.2%)		14 (18.2%)	10 (13.3%)	13 (17.3%)	10 (15.9%)	
Head and neck	2 (4.2%)	0 (0.0%)	0 (0.0%)	2 (3.3%)		10 (13.0%)	4 (5.3%)	1 (1.3%)	1 (1.6%)	
Uro-gynecologic	12 (25.0%)	8 (16.7%)	14 (29.2%)	13 (21.3%)		28 (36.4%)	21 (28.0%)	25 (33.3%)	27 (42.9%)	
**Comorbidity**					0.878					0.360
No	21 (43.8%)	24 (50.0%)	25 (52.1%)	31 (50.8%)		27 (35.1%)	31 (41.3%)	25 (33.3%)	16 (25.4%)	
Yes	27 (56.3%)	24 (50.0%)	23 (47.9%)	30 (49.2%)		50 (64.9%)	44 (58.7%)	50 (66.7%)	47 (74.6%)	
**NRS-2002**					0.184					**0.048**
<3	38 (80.9%)	41 (87.2%)	45 (93.8%)	58 (95.1%)		55 (74.3%)	62 (84.9%)	60 (81.1%)	59 (93.7%)	
>=3	9 (19.1%)	6 (12.8%)	3 (6.3%)	3 (4.9%)		19 (25.7%)	11 (15.1%)	14 (18.9%)	4 (6.3%)	
**SARC-F**					**<0.001**					0.134
<4	28 (59.6%)	40 (85.1%)	45 (93.8%)	55 (93.2%)		56 (76.7%)	63 (86.3%)	63 (85.1%)	58 (92.1%)	
>=4	19 (40.4%)	7 (14.9%)	3 (6.3%)	4 (6.8%)		17 (23.3%)	10 (13.7%)	11 (14.9%)	5 (7.9%)	
**Weight (kg)**	71.1 (18.2)	67.4 (16.5)	68.0 (12.8)	68.3 (12.6)	0.878	78.7 (17.9)	80.0 (14.0)	79.1 (15.1)	81.0 (15.0)	0.612

**BMI (kg/m²)**					0.857					0.768
Normal-weight	14 (29.8%)	20 (42.6%)	21 (43.8%)	24 (39.3%)		26 (35.1%)	21 (28.8%)	27 (36.5%)	15 (23.8%)	
Overweight	16 (34.0%)	16 (34.0%)	15 (31.3%)	21 (34.4%)		29 (39.2%)	34 (46.6%)	31 (41.9%)	29 (46.0%)	
Obese	17 (36.2%)	11 (23.4%)	12 (25.0%)	16 (26.2%)		19 (25.7%)	18 (24.7%)	16 (21.6%)	19 (30.2%)	
**WC (cm)**	94.2 (15.3)	87.8 (16.7)	89.6 (12.1)	89.3 (12.9)	0.427	101.0 (14.0)	98.8 (10.5)	99.8 (12.5)	100.2 (11.0)	0.884
**HC (cm)**	104.6 (13.9)	96.9 (13.5)	101.0 (11.5)	101.8 (10.9)	0.293	102.3 (11.2)	102.2 (10.4)	101.4 (10.2)	103.1 (10.2)	0.612
**PhA (°)**	5.0 (1.1)	5.6 (0.9)	5.3 (1.0)	5.8 (1.0)	**0.015**	5.3 (1.4)	5.8 (0.9)	5.8 (1.4)	6.1 (1.3)	**0.003**
**ECW (L)**	17.1 (3.0)	15.9 (2.3)	16.8 (3.7)	15.9 (2.3)	0.332	21.7 (3.8)	21.4 (3.8)	21.0 (4.2)	21.0 (3.7)	0.667
**ICW (L)**	16.4 (4.2)	17.4 (2.9)	16.7 (3.1)	17.9 (2.5)	0.142	21.9 (5.7)	23.9 (3.7)	23.2 (4.6)	25.2 (5.3)	**0.001**
**BCMI (kg/m²)**	8.7 (2.2)	9.3 (1.4)	8.7 (1.6)	9.6 (1.1)	**0.042**	10.1 (2.6)	10.9 (1.7)	10.7 (2.1)	11.6 (2.2)	**0.001**
**FMI (kg/m²)**	10.8 (5.0)	8.8 (4.5)	8.7 (3.2)	8.8 (3.7)	0.283	7.3 (3.4)	6.6 (2.6)	7.0 (3.5)	6.4 (2.5)	0.847
**FFMI (kg/m²)**	18.2 (2.6)	18.0 (1.9)	17.6 (1.8)	18.3 (1.2)	0.188	20.4 (2.9)	20.9 (2.1)	20.5 (2.2)	21.4 (2.4)	0.065
**SMI (kg/m²)**	7.7 (1.4)	7.7 (1.0)	7.7 (1.0)	7.9 (0.7)	0.427	10.1 (1.5)	10.3 (1.1)	10.0 (1.3)	10.5 (1.5)	0.106
**ASMI (kg/m²)**	6.8 (1.3)	6.7 (1.0)	6.5 (0.9)	6.9 (0.7)	0.265	7.8 (1.3)	8.0 (0.9)	7.9 (1.0)	8.3 (1.2)	0.061
**MedDiet Score (%)**					0.878					0.884
<7	15 (31.3%)	12 (25.0%)	15 (31.3%)	19 (31.1%)		32 (41.6%)	31 (41.3%)	29 (38.7%)	22 (34.9%)	
≥7	33 (68.8%)	36 (75.0%)	33 (68.8%)	42 (68.9%)		45 (58.4%)	44 (58.7%)	46 (61.3%)	41 (65.1%)	
**SumSc**	71.4 (18.6)	74.0 (15.9)	80.0 (12.8)	81.3 (13.3)	**0.044**	71.7 (18.7)	82.5 (11.2)	85.9 (13.1)	89.9 (9.6)	**<0.001**
**Functional score**	67.8 (21.4)	72.0 (18.1)	78.9 (13.6)	79.2 (15.6)	0.053	70.4 (19.7)	81.2 (14.6)	86.3 (14.6)	89.9 (11.5)	**<0.001**
Physical	66.4 (26.2)	80.6 (17.7)	83.6 (15.7)	87.3 (13.0)	**0.001**	70.3 (24.9)	85.4 (14.1)	88.5 (15.0)	92.5 (10.4)	**<0.001**
Role	56.3 (37.3)	70.5 (28.8)	76.4 (24.5)	85.5 (20.3)	**0.001**	65.4 (35.9)	81.8 (23.6)	89.3 (22.2)	91.5 (17.9)	**<0.001**
Emotional	61.6 (27.9)	56.6 (27.3)	68.4 (24.6)	65.6 (24.4)	0.265	65.0 (27.5)	71.9 (23.5)	77.7 (19.5)	83.1 (20.0)	**<0.001**
Cognitive	83.7 (20.2)	78.1 (22.1)	87.5 (15.9)	80.1 (22.7)	0.283	80.7 (19.3)	88.0 (15.4)	88.7 (17.8)	93.1 (12.2)	**<0.001**

Social	71.2 (29.7)	74.3 (24.3)	78.5 (22.0)	77.3 (23.8)	0.796	70.6 (30.0)	78.9 (24.6)	87.3 (19.7)	89.4 (17.8)	**<0.001**
**GHS**	50.3 (24.7)	51.9 (24.8)	62.2 (19.1)	62.6 (22.1)	0.114	48.9 (28.1)	59.7 (21.7)	63.6 (20.9)	68.1 (24.5)	**<0.001**
**Symptom score**	25.4 (17.6)	24.5 (16.5)	19.1 (14.4)	17.7 (13.5)	0.129	27.6 (18.6)	16.6 (11.2)	14.2 (13.6)	10.2 (9.5)	**<0.001**
Fatigue	49.8 (28.9)	40.3 (25.7)	32.6 (23.0)	28.2 (21.0)	**0.009**	41.8 (28.1)	25.8 (20.5)	21.2 (21.1)	17.1 (20.4)	**<0.001**
Nausea and vomiting	15.3 (23.8)	10.4 (16.4)	6.3 (10.7)	4.9 (10.2)	0.142	12.6 (22.0)	4.4 (12.7)	5.6 (12.7)	4.0 (8.8)	**0.021**
Pain	40.3 (30.9)	31.9 (27.0)	22.9 (21.6)	21.0 (22.1)	**0.018**	32.3 (31.6)	20.9 (23.3)	15.1 (21.9)	11.9 (19.3)	**<0.001**
Dyspnea	21.5 (27.9)	25.7 (26.8)	16.0 (24.8)	10.9 (18.0)	0.077	24.2 (28.9)	12.4 (18.8)	14.7 (21.4)	6.3 (14.5)	**<0.001**
Insomnia	35.4 (30.3)	43.1 (29.9)	35.4 (34.7)	36.1 (28.7)	0.593	40.7 (34.0)	25.3 (27.3)	22.2 (25.3)	19.0 (24.5)	**<0.001**
Appetite loss	22.2 (28.6)	18.8 (26.5)	15.3 (24.8)	12.0 (22.8)	0.293	24.2 (33.2)	11.6 (22.9)	8.9 (20.7)	7.4 (18.4)	**0.002**
Constipation	22.9 (30.9)	19.4 (29.8)	19.4 (27.4)	20.2 (23.8)	0.878	27.7 (28.8)	22.2 (27.0)	19.1 (26.4)	10.6 (17.8)	**0.003**
Diarrhea	4.2 (11.1)	9.0 (19.1)	6.9 (16.8)	6.0 (16.7)	0.796	16.0 (26.3)	10.2 (25.1)	7.6 (17.8)	4.2 (11.2)	**0.021**
Financial difficulties	17.4 (27.5)	22.2 (28.6)	17.4 (27.5)	19.7 (23.1)	0.727	28.6 (31.4)	16.4 (26.5)	13.8 (22.7)	11.1 (16.9)	**0.002**

1Mean (SD); n (%)
2Kruskal-Wallis rank sum test; Pearson's Chi-squared test;
NRS-2002, Nutritional Risk Screening 2002; SARC-F, Strength, Assistance with walking, Rise from a chair, Climb stairs, and Falls; BMI, Body Mass Index; WC, Waist circumference; HC, Hip circumference; PhA, Phase angle; ECW, Extracellular water; ICW, Intracellular water; BCMI, Body cell mass index; FMI, Fat mass index; FFMI, Fat-free mass index; SMI, Skeletal muscle mass index; ASMI, Appendicular skeletal muscle mass index; SumSc, Summary Score; GHS, Global Health Score; MedDiet, Mediterranean Diet

Table S2 Multivariable-adjusted models for the association of physical activity levels with functional HRQoL scales.

	**Physical**		**Role**		**Emotional**		**Cognitive**		**Social**		**GHS**	
**MET levels**	**OR**	**95% CI[1]**	**OR**	**95% CI[1]**	**OR**	**95% CI[1]**	**OR**	**95% CI[1]**	**OR**	**95% CI[1]**	**OR**	**95% CI[1]**
Low	—	—	—	—	—	—	—	—	—	—	—	—
Moderate	2.57	1.58, 4.17*	2.31	1.41, 3.82*	1.04	0.65, 1.66	1.04	0.63, 1.72	1.33	0.81, 2.19	1.65	1.03, 2.64*
Moderate to High	3.94	2.43, 6.41*	3.96	2.37, 6.68*	1.71	1.07, 2.74*	1.51	0.92, 2.50	2.08	1.27, 3.43*	2.23	1.40, 3.58*
Intensive	4.97	3.02, 8.25*	5.52	3.21, 9.60*	2.06	1.28, 3.34*	1.39	0.83, 2.34	2.04	1.23, 3.40*	2.65	1.63, 4.32*
**NRS-2002**												
<3	—	—	—	—	—	—	—	—	—	—	—	—
≥3	0.35	0.22, 0.55*	0.33	0.20, 0.54*	0.52	0.32, 0.81*	0.82	0.51, 1.33	0.71	0.44, 1.15	0.34	0.21, 0.54*
**SARC-F**												
<4	—	—	—	—	—	—	—	—	—	—		
≥4	0.14	0.09, 0.23*	0.13	0.08, 0.21*	0.24	0.16, 0.38*	0.40	0.25, 0.62*	0.27	0.17, 0.43*	0.24	0.15, 0.37*
**p-trend**	**<0.001**		**<0.001**		**<0.001**		0.09		**0.001**		**<0.001**	

Table S3a Multivariable-adjusted models for the association of physical activity levels with symptom HRQoL scales

	Fatigue		Nausea and vomiting		Pain		Dyspnea		Insomnia	
**Physical quartiles**	**OR**	**95% CI[1]**	**OR**	**95% CI[1]**	**OR**	**95% CI[1]**	**OR**	**95% CI[1]**	**OR**	**95% CI[1]**
Low	—	—	—	—	—	—	—	—	—	—
Moderate	0.41	0.25, 0.66*	0.47	0.25, 0.85*	0.55	0.34, 0.90*	1.00	0.58, 1.72	0.75	0.46, 1.23
Moderate to High	0.29	0.17, 0.47*	0.52	0.28, 0.94*	0.38	0.23, 0.62*	0.71	0.41, 1.22	0.54	0.33, 0.90*
Intensive	0.23	0.14, 0.37*	0.40	0.21, 0.74*	0.31	0.18, 0.52*	0.37	0.20 0.68*	0.58	0.35, 0.95*
**Score NRS2002**										
< 3	—	—	—	—	—	—	—	—	—	—
>= 3	3.09	1.97, 4.85*	2.77	1.61, 4.75*	2.13	1.35, 3.38*	1.68	1.01, 2.78*	1.57	0.97, 2.53
**Score SARCF**										
.< 4	—	—	—	—	—	—	—	—	—	—
>= 4	5.80	3.71, 9.11*	3.01	1.81, 5.02*	4.05	2.60, 6.34*	2.84	1.75, 4.64*	1.54	0.98, 2.41
**p-trend**	**<0.001**		**0.008**		**<0.001**		**<0.001**		**0.015**	

Table S3b. Multivariable-adjusted models for the association of physical activity levels with symptom HRQoL scales

	Appetite loss		Constipation		Diarrhoea		Financial difficulties	
**Physical quartiles**	**OR**	**95% CI[1]**	**OR**	**95% CI[1]**	**OR**	**95% CI[1]**	**OR**	**95% CI[1]**
Low	—	—	—	—	—	—	—	—
Moderate	0.49	0.27, 0.87*	0.75	0.45, 1.26	0.66	0.34, 1.27	0.64	0.37, 1.08
Moderate to High	0.41	0.23, 0.75*	0.58	0.34, 0.97*	0.63	0.33, 1.21	0.57	0.33, 0.96*
Intensive	0.37	0.20, 0.70*	0.61	0.36, 1.04	0.49	0.24, 0.99*	0.68	0.40, 1.15
**Score NRS2002**								
< 3	—	—	—	—	—	—	—	—
>= 3	4.54	2.72, 7.61*	2.12	1.31, 3.46*	1.15	0.63, 2.09	1.46	0.89, 2.39
**Score SARCF**								
< 4	—	—	—	—	—	—	—	—
>= 4	2.62	1.59, 4.32*	1.17	0.72, 1.88	1.71	0.97, 2.97	2.42	1.51, 3.88*
**p-trend**	**0.001**		**0.043**		0.055		0.134	

Table S4a. Multivariable-adjusted models for the association of physical activity levels with symptom HRQoL scales stratified by gender

	**Physical**				**Role**				**Emotional**			
	**Male**		**Female**		**Male**		**Female**		**Male**		**Female**	
**Physical quartiles**	**OR**	**95% CI[1]**	**OR**	**95% CI[1]**	**OR**	**95% CI[1]**	**OR**	**95% CI[1]**	**OR**	**95% CI[1]**	**OR**	**95% CI[1]**
Low	—	—	—	—	—	—	—	—	—	—	—	—
Moderate	3.03	1.63, 5.68*	1.80	0.81, 3.99	2.57	1.36, 4.90*	1.79	0.80, 4.01	1.47	0.81, 2.67	0.55	0.25, 1.20
Moderate to High	5.60	2.98, 10.69*	2.87	1.33, 6.24*	6.00	2.96, 12.54*	2.42	1.10, 5.35*	2.03	1.11, 3.74*	1.42	0.67, 3.03
Intensive	6.90	3.48, 13.97*	3.40	1.62, 7.23*	6.23	2.90, 13.93*	4.76	2.17, 10.62*	3.65	1.89, 7.11*	1.01	0.48, 2.12
**Score NRS2002**												
< 3	—	—	—	—	—	—	—	—	—	—	—	—
>= 3	0.33	0.19, 0.60*	0.40	0.18, 0.89*	0.34	0.18, 0.62*	0.34	0.15, 0.78*	0.53	0.31, 0.92*	0.48	0.22, 1.05
**Score SARCF**												
< 4	—	—	—	—	—	—	—	—	—	—	—	—
>= 4	0.17	0.09, 0.33*	0.13	0.06, 0.27*	0.15	0.08, 0.28*	0.11	0.05, 0.24*	0.21	0.12, 0.38*	0.29	0.14, 0.59*
**p-trend**	**<0.001**		**<0.001**		**<0.001**		**<0.001**		**<0.001**		0.40	

Table S4b. Multivariable-adjusted models for the association of physical activity levels with functional HRQoL scales stratified by gender

	Cognitive				Social				GHS			
	**Male**		**Female**		**Male**		**Female**		**Male**		**Female**	
**Physical quartiles**	**OR**	**95% CI[1]**	**OR**	**95% CI[1]**	**OR**	**95% CI[1]**	**OR**	**95% CI[1]**	**OR**	**95% CI[1]**	**OR**	**95% CI[1]**
Low	—	—	—	—	—	—	—	—	—	—	—	—
Moderate	2.07	1.09, 3.98*	0.35	0.15, 0.80*	1.71	0.91, 3.25	0.87	0.39, 1.92	2.25	1.24, 4.11*	0.90	0.41, 1.97
Moderate to High	2.23	1.17, 4.28*	0.95	0.42, 2.14	3.35	1.73, 6.54*	1.23	0.57, 2.68	2.69	1.47, 4.96*	1.74	0.82, 3.70
Intensive	3.70	1.80, 7.81*	0.41	0.18, 0.91*	3.69	1.82, 7.64*	1.00	0.47, 2.15	4.10	2.12, 8.01*	1.50	0.72, 3.15
**Score NRS2002**												
< 3	—	—	—	—	—	—	—	—	—	—	—	—
>= 3	0.80	0.45, 1.46	0.86	0.37, 1.94	0.48	0.26, 0.86*	1.38	0.62, 3.15	0.31	0.18, 0.55*	0.34	0.15, 0.75*
**Score SARCF**												
< 4	—	—	—	—	—	—	—	—	—	—	—	—
>= 4	0.42	0.23, 0.75*	0.43	0.20, 0.89*	0.19	0.10, 0.35*	0.48	0.23, 0.98*	0.23	0.13, 0.40*	0.24	0.11, 0.49*
**p-trend**	**<0.001**		0.19		**<0.001**		0.75		**<0.001**		0.11	

Table S5a Multivariable-adjusted models for the association of physical activity levels with symptoms HRQoL scales stratified by gender

	**Fatigue**				**Nausea and vomiting**				**Pain**			
	**Male**		**Female**		**Male**		**Female**		**Male**		**Female**	
**Physical quartiles**	**OR**	**95% CI[1]**	**OR**	**95% CI[1]**	**OR**	**95% CI[1]**	**OR**	**95% CI[1]**	**OR**	**95% CI[1]**	**OR**	**95% CI[1]**
Low	—	—	—	—	—	—	—	—	—	—	—	—
Moderate	0.32	0.17, 0.59*	0.69	0.32, 1.52	0.28	0.12, 0.65*	0.99	0.41, 2.39	0.49	0.26, 0.92*	0.75	0.35, 1.63
Moderate to High	0.23	0.12, 0.43*	0.39	0.18, 0.84*	0.46	0.21, 1.02	0.70	0.29, 1.74	0.34	0.17, 0.66*	0.44	0.20, 0.97*
Intensive	0.17	0.08, 0.33*	0.34	0.16, 0.73*	0.42	0.17, 0.99*	0.49	0.20, 1.21	0.26	0.12, 0.52*	0.38	0.18, 0.80*
**Score NRS2002**												
< 3	—	—	—	—	—	—	—	—	—	—	—	—
>= 3	3.13	1.79, 5.48*	3.20	1.47, 7.00*	2.80	1.40, 5.61*	2.37	0.95, 5.82	2.20	1.25, 3.91*	2.13	0.97, 4.76
**Score SARCF**												
< 4	—	—	—	—	—	—	—	—	—	—	—	—
>= 4	5.60	3.13, 10.07*	5.21	2.54, 10.83*	3.47	1.77, 6.79*	1.94	0.84, 4.46	3.76	2.11, 6.71*	4.35	2.12, 8.97*
**p-trend**	**<0.001**		**0.002**		**0.012**		0.06		**<0.001**		**0.05**	

Table S5b. Multivariable-adjusted models for the association of physical activity levels with symptoms HRQoL scales stratified by gender

	Dyspnoea				Insomnia				Appetite loss			
	**Male**		**Female**		**Male**		**Female**		**Male**		**Female**	
**Physical quartiles**	**OR**	**95% CI[1]**	**OR**	**95% CI[1]**	**OR**	**95% CI[1]**	**OR**	**95% CI[1]**	**OR**	**95% CI[1]**	**OR**	**95% CI[1]**
Low	—	—	—	—	—	—	—	—	—	—	—	—
Moderate	0.51	0.24, 1.04	2.86	1.19, 7.07*	0.37	0.19, 0.70*	2.21	1.00, 4.94	0.33	0.15, 0.74*	0.97	0.40, 2.36
Moderate to High	0.50	0.24, 1.02	1.08	0.44, 2.70	0.33	0.17, 0.62*	1.07	0.47, 2.41	0.25	0.10, 0.58*	0.77	0.31, 1.90
Intensive	0.24	0.09, 0.57*	0.69	0.28, 1.72	0.28	0.13, 0.55*	1.47	0.70, 3.14	0.25	0.09, 0.62*	0.60	0.25, 1.47
**Score NRS2002**												
**< 3**	—	—	—	—	—	—	—	—	—	—	—	—
**>= 3**	2.09	1.12, 3.90*	1.22	0.49, 2.99	1.71	0.95, 3.08	1.35	0.58, 3.12	4.01	2.07, 7.84*	5.95	2.51, 14.47*
**Score SARCF**												
**< 4**	—	—	—	—	—	—	—	—	—	—	—	—
**>= 4**	3.15	1.67, 5.98*	2.52	1.12, 5.70*	1.90	1.06, 3.39*	0.94	0.45, 1.94	3.44	1.76, 6.72*	1.49	0.68, 3.25
**p-trend**	**0.002**		0.15		**<0.001**		0.71		**0.003**		0.22	

Table S5c Multivariable-adjusted models for the association of physical activity levels with symptoms HRQoL scales stratified by gender

	**Constipation**				**Diarrhoea**				**Financial difficulties**			
	**Male**		**Female**		**Male**		**Female**		**Male**		**Female**	
**Physical quartiles**	**OR**	**95% CI[1]**	**OR**	**95% CI[1]**	**OR**	**95% CI[1]**	**OR**	**95% CI[1]**	**OR**	**95% CI[1]**	**OR**	**95% CI[1]**
Low	—	—	—	—	—	—	—	—	—	—	—	—
Moderate	0.69	0.36, 1.33	0.76	0.32, 1.81	0.40	0.17, 0.88*	2.22	0.61, 8.04	0.38	0.19, 0.74*	1.58	0.65, 3.90
Moderate to High	0.46	0.23, 0.88*	0.76	0.32, 1.79	0.41	0.18, 0.89*	2.00	0.55, 7.31	0.37	0.18, 0.72*	1.13	0.45, 2.82
Intensive	0.31	0.15, 0.66*	1.16	0.52, 2.60	0.27	0.10, 0.66*	1.92	0.52, 7.18	0.30	0.14, 0.62*	2.02	0.88, 4.83
**Score NRS2002**												
< 3	—	—	—	—	—	—	—	—	—	—	—	—
>= 3	2.19	1.19, 4.04*	1.90	0.82, 4.42	1.26	0.62, 2.51	0.86	0.23, 3.13	1.33	0.71, 2.45	2.08	0.88, 4.91
**Score SARCF**												
< 4	—	—	—	—	—	—	—	—	—	—	—	—
>= 4	1.17	0.62, 2.17	1.22	0.56, 2.62	2.15	1.09, 4.22*	1.18	0.40, 3.48	3.11	1.67, 5.86*	2.02	0.94, 4.37
**p-trend**	**0.001**		0.73		**0.009**		0.37		**0.002**		0.19	
